# A Novel Microtubule-Tau Association Enhancer and Neuroprotective Drug Candidate: Ac-SKIP

**DOI:** 10.3389/fncel.2019.00435

**Published:** 2019-10-01

**Authors:** Yanina Ivashko-Pachima, Illana Gozes

**Affiliations:** Dr. Diana and Zelman Elton (Elbaum) Laboratory for Molecular Neuroendocrinology, Department of Human Molecular Genetics and Biochemistry, Sackler Faculty of Medicine, Sagol School of Neuroscience, Adams Super Center for Brain Studies, Tel Aviv University, Tel Aviv, Israel

**Keywords:** tau, microtubules, EBs, ADNP, SKIP

## Abstract

Activity-dependent neuroprotective protein (ADNP) has been initially discovered through its eight amino acid sequence NAPVSIPQ, which shares SIP motif with SALLRSIPA – a peptide derived from activity-dependent neurotrophic factor (ADNF). Mechanistically, both NAPVSIPQ and SALLRSIPA contain a SIP motif that is identified as a variation of SxIP domain, providing direct interaction with microtubule end-binding proteins (EBs). The peptide SKIP was shown before to provide neuroprotection *in vitro* and protect against Adnp-related axonal transport deficits *in vivo*. Here we show, for the first time that SKIP enhanced microtubule dynamics, and prevented Tau-microtubule dissociation and microtubule disassembly induced by the Alzheimer’s related zinc intoxication. Furthermore, we introduced, CH_3_CO-SKIP-NH_2_ (Ac-SKIP), providing efficacious neuroprotection. Since microtubule – Tau organization and dynamics is central in axonal microtubule cytoskeleton and transport, tightly related to aging processes and Alzheimer’s disease, our current study provides a compelling molecular explanation to the *in vivo* activity of SKIP, placing SKIP motif as a central focus for MT-based neuroprotection in tauopathies with axonal transport implications.

## Introduction

Activity-dependent neuroprotective protein (ADNP) has been initially discovered through its eight amino acid sequence NAPVSIPQ (Bassan et al., 1999), which shares SIP motif with SALLRSIPA – a peptide derived from activity-dependent neurotrophic factor (ADNF) (Brenneman and Gozes, 1996; Zamostiano et al., 1999). ADNF, and then ADNP, have been originally found to mediate neuroprotective and neurotrophic activities of the vasoactive intestinal peptide (VIP) (Brenneman and Gozes, 1996; Brenneman et al., 1998). Subsequent studies have shown that ADNP is dysregulated in schizophrenia (Dresner et al., 2011; Merenlender-Wagner et al., 2015) and Alzheimer’s disease (AD) (Yang et al., 2012; Malishkevich et al., 2016), and mutated in autism spectrum disorder (ASD) with 0.17% prevalence (together, these ASD cases are now identified as the ADNP syndrome) (Helsmoortel et al., 2014; Gozes et al., 2015). Importantly, it has been shown that ADNP is the only down-regulated protein in the serum of AD patients (Yang et al., 2012) and expression levels of ADNP in plasma/serum and lymphocyte is correlated with AD clinical progression, disease pathology and premorbid intelligence (Malishkevich et al., 2016). Animal studies with mice expressing *Adnp* from only one allele (Adnp^±^) have shown that *Adnp* deficiency is associated with age-dependent neurodegeneration and cognitive impairment, coupled with tauopathy-like features such as an increase formation of tangle-like structures, defective axonal transport, and Tau hyperphosphorylation (Vulih-Shultzman et al., 2007).

Both peptides NAPVSIPQ (ADNP-derived) and SALLRSIPA (ADNF-derived) have shown neuroprotective activities against cognitive decline and peripheral neuropathy in various animal models (Shiryaev et al., 2011; Gozes et al., 2016). NAP biochemical activity has been broadly examined and found to be inextricably linked with microtubules (MTs) and MT-related cellular events: NAP increases MT elongation and dynamics (Ivashko-Pachima et al., 2017), augments axonal transport, in the face of MT deficiencies (Jouroukhin et al., 2013), and protects Tau-MT association under various insults (Oz et al., 2012; Ivashko-Pachima et al., 2017). Mechanistically, both NAPVSIPQ and SALLRSIPA contain a SIP motif that is identified as a variation of SxIP domain, providing direct interaction with MT end-binding proteins (EBs) (Honnappa et al., 2009). Our initial studies have shown a direct interaction of SIP- and SKIP-containing peptides with EB1 and EB3 proteins (Oz et al., 2014). We have further shown that four amino acid peptide SKIP docks to the EB3 binding site *in silico*, and stimulates axonal transport *in vivo*, which is reduced as a consequence of *Adnp* deficiency in Adnp^±^ mice (Amram et al., 2016).

Here, we aimed to test the activity of SKIP and modified SKIP – CH_3_CO-SKIP-NH_2_ (Ac-SKIP) on MT dynamics and integrity, mediated by MT-associated proteins EB1 and Tau. EB proteins can directly influence MT dynamics (Komarova et al., 2009) and also enroll other MT-affecting proteins to the growing MT plus-ends (Honnappa et al., 2009). Tau is a broadly known MT-associated protein which stimulates MT assembly and Tau physiological and biochemical impairments are well-studied in a variety of neurodegenerative diseases, referred to tauopathies (Kneynsberg et al., 2017). Furthermore, it has been found that Tau directly associates with EB1 and EB3 proteins and modulates their location on the MTs (Sayas et al., 2015). Here, we tested different concentrations of SKIP and Ac-SKIP and found that at 10^–9^ M SKIP and Ac-SKIP exhibited consistent and significant activity: (1) increased elongation of freshly growing MT plus-ends; and prevented, (2) Tau-MT dissociation, and (3) MT disassembly, induced by extracellular zinc. Thus, our current study provided a molecular explanation to the previously observed effect of SKIP on MT-related functions: stimulation of axonal transport and normalization of social memory in Adnp ± mice. Furthermore, our results showed that Ac-SKIP provided surprisingly more efficacious neuroprotection and suggested that SKIP might be the shortest motif essential for MT-based neuroprotection, mediated by EB proteins and Tau.

## Materials and Methods

### Cell Culture and Treatments

Mouse neuroblastoma N1E-115 cells (ATCC, Bethesda, MD, United States) were maintained in Dulbecco’s modified Eagle’s medium (DMEM), 10% fetal bovine serum (FBS), 2 mM glutamine and 100 U/ml penicillin, 100 mg/ml streptomycin (Biological Industries, Beit Haemek, Israel). Human neuroblastoma SH-SYS5 cells (ECACC, Public Health England, Porton Down, Salisbury, United Kingdom; passage numbers from 14 to 16) were maintained in Ham’s F12: minimum essential media (MEM) Eagle (1:1), 2 mM Glutamine, 1% non-essential amino acids, 15% FBS and 100 U/ml penicillin, 100 mg/ml streptomycin (Biological Industries, Beit Haemek, Israel). Cells were incubated in 95% air/5% CO_2_ in a humidified incubator at 37°C. Cells were differentiated with reduced FBS (2%) and DMSO (1.25%) containing medium (N1E-115 cells) or with retinoic acid at a concentration of 10 μM (SH-SY5Y cells) during 7 days before each experiment. Differentiated N1E-115 cells were treated for 2 or 4 h with SKIP/Ac-SKIP in final concentrations of 10^–12^ – 10^–6^ M, in the absence or presence of zinc (400 μM of ZnCl_2_, stock solution – 0.1 M ZnCl_2_ in water, Sigma, Rehovot, Israel).

### Cell Viability Assay

A week before the experiment, N1E-115 cells were plated onto 96-well plates at a concentration of 5000 cells/well in 100 μl of the growth medium, which was replaced by differentiation medium a day after cell seeding. On an experimental day, cells were treated during 4 h with 400 μM of ZnCl_2_ in the absence or presence of NAP (10^–12^ – 10^–9^ M). Cell survival was measured using XTT-based cell proliferation kit (Biological Industries, Beit Haemek, Israel), which was performed according to the manufacturer’s instructions. The absorbance of the samples was measured with a spectrophotometer (ELISA reader) at wavelengths of 490/630 nm.

### Time-Lapse Imaging of EB1 Comet-Like Structures

N1E-115 cells were plated on 35 mm dishes (81156, μ-Dish, Ibidi, Martinsried, Germany) at a concentration of 25000 cells/dish and then differentiated with reduced FBS, DMSO-containing medium during seven days. 48 h before live imaging, differentiated N1E-115 cells were transfected with 1 μg of EB1-RFP expressing plasmid. On an experimental day, N1E-115 cells were incubated at 37°C with a 5% CO_2_/95% air mixture in a thermostatic chamber placed on the stage of a Leica TCS SP5 confocal microscope (objective ×100 (PL Apo) oil immersion, NA 1.4). Time-lapse images were automatically captured every 3 s during 2 min, using the Leica LAS AF software (Leica Microsystems, Wetzlar, Germany). Data were collected and analyzed by Imaris software (Bitplane, Concord, MA, United States).

### Fluorescence Recovery After Photobleaching

Forty-eight hours before a fluorescence recovery after photobleaching (FRAP) experiment, differentiated N1E-115 cells were transfected with a 1 μg pmCherry-C1-Tau3R plasmid (Ivashko-Pachima et al., 2019). FRAP was performed using a Leica TCS SP5 confocal microscope [objective 100× (PL Apo) oil immersion, NA 1.4]. ROIs (regions of interest) for photobleaching were drawn in the proximal cell branches. mCherry-Tau molecules were bleached with argon laser during 15 s, and data about fluorescence recovery after bleaching were automatically collected (80 images every 0.74 s) by the Leica LAS AF software. Fluorescence intensities were measured by ImageJ Fiji (Schindelin et al., 2012), obtained data were normalized with easyFRAP software (Rapsomaniki et al., 2012). FRAP recovery results were fitted by a one-phase exponential association function and recovery curves were built using GraphPad Prism 6 (GraphPad Software, Inc., La Jolla, CA). Samples with *R*^2^ < 0.9 were excluded.

### Polymerized vs. Soluble Tubulin Quantification Assay

Quantification of tubulin polymerization was performed as previously (Oz et al., 2012; Ivashko-Pachima et al., 2017; Ivashko-Pachima and Gozes, 2018). Briefly, in order to extract soluble tubulin (S) differentiated N1E-115 cells were lysed with TritonX-100-containing MT-buffer (80 mM PIPES pH6.8, 1 mM MgCl_2_, 2 mM EGTA, 5% Glycerol, with or without 0.5% TritonX-100) at room temperature for 5 min while centrifuging at 800 rcf; in order to collect the polymerized tubulin (P) pelleted cells were rinsed once again with equal volume of modified RIPA buffer (50 mM Tris–HCl pH7.4, 150 mM NaCl, 2 mM EGTA, 1% TritonX-100, 0.1% SDS, 0.1% sodium Deoxycholate, protease and phosphatase inhibitors: 1 mM phenylmethylsulphonyl-fluoride (PMSF), leupeptin 25 μg/ml, pepstatin 25 μg/ml, Na_3_VO_4_ 1 mM, NaF 20 mM) on ice. The soluble and polymerized tubulin fractions were each mixed with the same amount of sample buffer (10 mM Tris–HCl, pH6.8, 1.5% SDS, 0.6% DTT and 6% (v/v) glycerol) and heated at 95°C for 5 min. An equal volume of each fraction was analyzed by immunoblotting with appropriate antibodies, and the results following ECL development (by a chemiluminescence kit, Pierce, Rockford, IL, United States) were quantified by densitometry (using GelQuant.NET software provided by biochemlabsolutions.com).

### Co-immunoprecipitation Assay

Proteins were extracted from differentiated human neuroblastoma SH-SY5Y cells and Co-IP assay was performed as previously reported (Merenlender-Wagner et al., 2015; Ivashko-Pachima et al., 2017) using Co-IP kit according to the manufacture protocol (Pierce, Rockford, IL, United States). Briefly, 10 μg of antibodies of interest (EB1, ab53358, Abcam, Cambridge, United Kingdom; and total Tau antibody, AT-5004, MBL, Billerica, MA, United States) were cross-linked to the 30 μl of A/G PLUS-agarose beads (provided by the Co-IP kit). 2 μg of SKIP or Ac-SKIP, diluted into lysis buffer were added per sample (1 mg of cell lysate) for 2 h at room temperature. Flow-through, wash and elution fractions were then collected and analyzed by immunoblotting with appropriate antibodies, and the results following ECL (Pierce) development were quantified by densitometry (GelQuant.NET software provided by biochemlabsolutions.com).

### Antibody List

Tubulin – monoclonal α-tubulin antibody (mouse IgG1 isotype, T6199, Sigma, Rehovot, Israel). Actin – mouse monoclonal actin antibody (Sigma, Rehovot, Israel). The secondary antibodies were peroxidase-conjugated AffiniPure goat anti-mouse IgG (Jackson ImmunoResearch, United States).

### Statistical Analysis

Data are presented as the mean ± SEM from at least 3 independent experiments performed in triplicates. Statistical analysis of the data was performed by using one-way ANOVA test (followed by the Turkey or LSD *post hoc* test) by IBM SPSS Statistics software version 23 (IBM, Armonk, NY, United States). ^∗^ *P* < 0.05, ^∗∗^ *P* < 0.01, ^∗∗∗^ *P* < 0.001. Detailed statistical data are summarized in the Supplementary File.

## Results

### The Protective Effect of SKIP and Ac-SKIP Against Cell Death Induced by Zinc Toxicity

**FIGURE 1:**
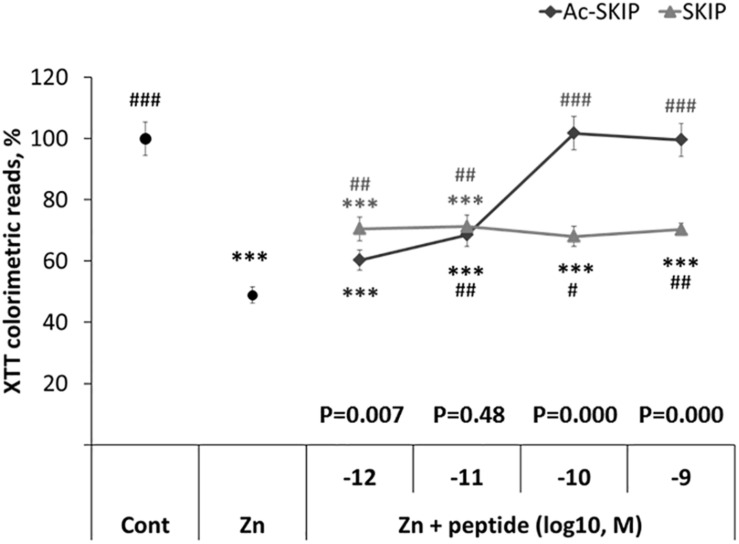
SKIP and Ac-SKIP exhibit protective activity against zinc cytotoxicity in differentiated neuroblastoma N1E-115 cells. Results of mitochondrial activity were obtained by XTT colorimetric assay. “Cont” – cells without any treatment (*n* = 60); “Zn” – cells, exposed to 400 μM of zinc (*n* = 58); “Zn + peptide” – cells treated with zinc (400 μM) and different concentrations of Ac-SKIP or SKIP: 10^–12^ M Ac-SKIP (*n* = 22)/SKIP (*n* = 14), 10^–11^ M Ac-SKIP (*n* = 22)/SKIP (*n* = 14), 10^–10^ M Ac-SKIP (*n* = 22)/SKIP (*n* = 14), 10^–9^ M Ac-SKIP (*n* = 22)/SKIP (*n* = 14). Statistical analysis was performed by One-way ANOVA: ^∗∗∗^*p* < 0.001, *post hoc* comparisons made in reference to “Cont” group; #*p* < 0.05, ##*p* < 0.01, ###*p* < 0.001, *post hoc* comparisons made in reference to “Zn” group. Statistical analysis within the concentration group of peptides was done by the Student’s *t*-test and *P*-values are displayed on the graph above concentration scale of peptides.

A colorimetric method for cell viability (based on the tetrazolium salt – XTT) was used to assess the protective activity of SKIP and Ac-SKIP at different concentrations (10^–12^ – 10^–9^M) against the cytotoxic effect of zinc in order to choose potent concentration for the further experiments. Differentiated neuroblastoma N1E-115 cells were treated with zinc alone or together with Ac-SKIP or SKIP for 4 h, and XTT-produced soluble dye was then measured by ELISA reader. Previously published data have shown a consistent and significant effect of zinc cytotoxicity at 400 μM (Sanchez-Martin et al., 2010; Oz et al., 2012; Ivashko-Pachima et al., 2017). Hence, here, we worked with 400 μM of zinc. Treatment with zinc caused a reduction of ∼50% in cell viability, and peptide treatments showed significant protection against cell death, except for Ac-SKIP at 10^–12^ M. SKIP treatment at every examined concentration exhibited nearly equal ∼20% protection that was found statistically significant. Ac-SKIP protective potency was found to be positively dependent on the peptide concentration: non-significant slight protection at 10^–12^ M; significant, a moderate effect at 10^–11^ M; and a ∼100% protective effect at 10^–10^ M and 10^–9^ M. Ac-SKIP and SKIP exhibited the same protective potency at 10^–11^ M and significantly different magnitude of protective activity at 10^–12^ M (with a greater effect of SKIP), 10^–10^ M and 10^–9^ M (with a significantly greater effect of Ac-SKIP). For further experiments, we chose to work with 10^–12^ M and 10^–9^ M of Ac-SKIP/SKIP (Figure 1).

### Ac-SKIP and SKIP Affect MT Dynamics

**FIGURE 2:**
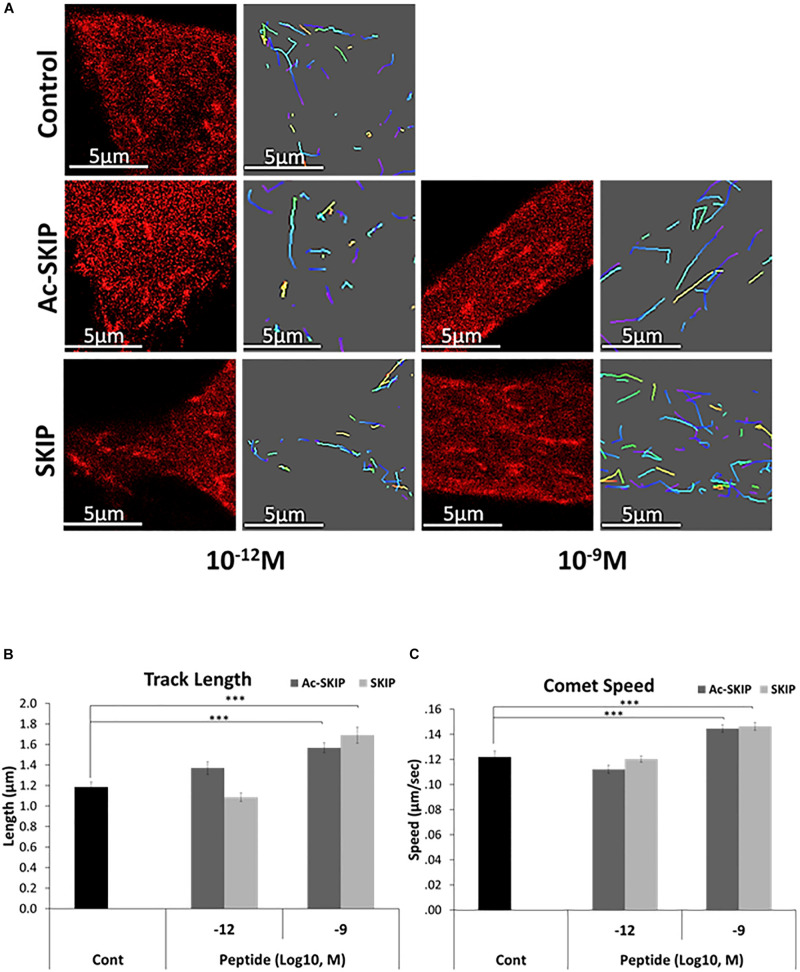
SKIP and Ac-SKIP affect MT dynamics. **(A)** Live imaging of N1E-115 cells expressing EB1-RFP was performed after 4 h of treatment with Ac-SKIP or SKIP at 10^–12^ M and 10^–9^ M. Transfected cells without peptide treatment were used as a control group (“Control”). Time-lapse images were automatically captured every 3 s. during a 1 min using the Leica LAS AF software. Tracks of EB1 comet like structures presented as colored lines and were obtained by the Imaris software. Graphs represent quantification of the average track length **(B)** and comet speed **(C)**. Data were collected in unbiased fashion by the Imaris software, and statistical analysis of the data was performed by One-way ANOVA. Statistical significance is represented by ^∗∗∗^*P* < 0.001. Comet length: Control, *n* = 46; Ac-SKIP 10^–12^ M, *n* = 29; SKIP 10^–12^ M, *n* = 20; Ac-SKIP 10^–9^ M, *n* = 62; SKIP 10^–9^ M, *n* = 43. Comet speed: Control, *n* = 47; Ac-SKIP 10^–12^ M, *n* = 30; SKIP 10^–12^ M, *n* = 20; Ac-SKIP 10^–9^ M, *n* = 65; SKIP 10^–9^ M, *n* = 43.

The direct interaction between SKIP and EB1/3 has been previously predicted by Pymol (Schrödinger, 2015), and peptides containing SxIP motif have displayed the association with EB1/3 in sulfolink columns. Here, we aimed to evaluate the effect of SKIP and Ac-SKIP on MT dynamics, mediated by direct interaction of these peptides with the EB1 protein. Differentiated neuroblastoma cells were subjected to transient transfection with expression plasmid encoding to EB1 protein, tagged to RFP (Figure 2A and Supplementary Figure S1). Single-cell time-lapse imaging allowed the evaluation of the effect of the peptides on MT dynamics by tracking RFP-EB1 comet-like structures decorating newly polymerized MT plus-ends. Time-lapse imaging followed by the 4 h peptide treatment with Ac-SKIP or SKIP showed that both Ac-SKIP and SKIP at 10^–9^ M (but not at 10^–12^ M) significantly augmented the track length (Figure 2B) and velocity (Figure 2C) of the EB1 comets, reflecting the lengths of the MT growing events and the speed of MT assembly, respectively. Thus, both peptides had an impact on MT dynamics.

### Protective Effect of Ac-SKIP and SKIP on Tau-MT Association, Disrupted by Zinc

**FIGURE 3:**
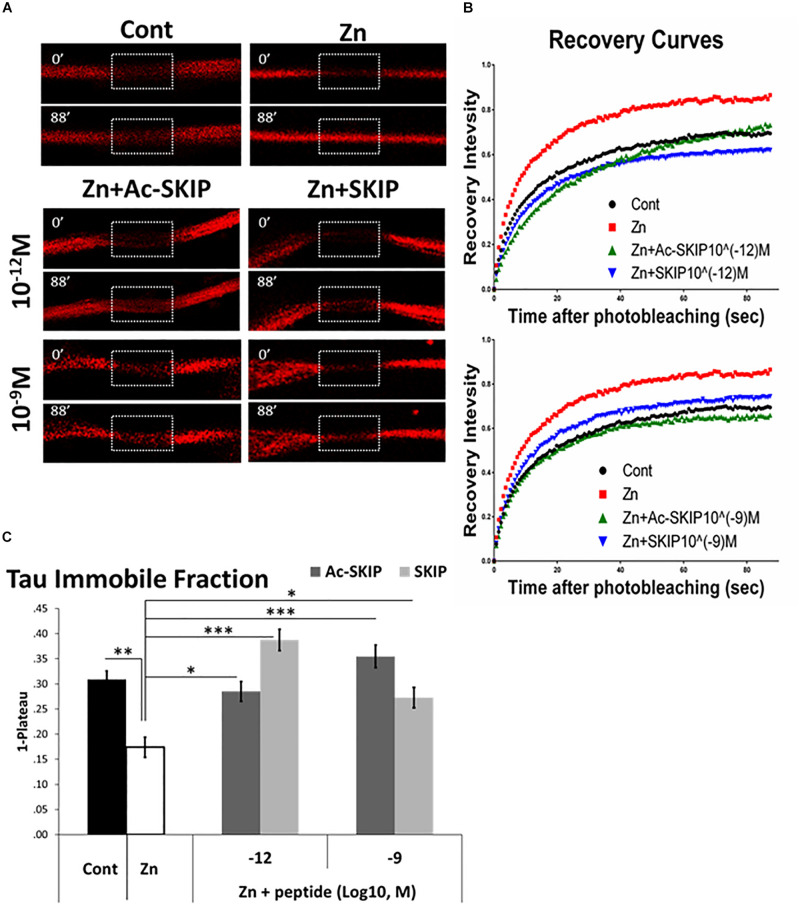
Ac-SKIP and SKIP protective effect on Tau interaction with MTs, assessed by FRAP analysis. FRAP was performed on differentiated neuroblastoma N1E-115 cells after 2 h of treatment with extracellular zinc (400 μM) alone or together with Ac-SKIP or SKIP (10^–12^ M and 10^–9^ M). **(A)** Representative images of FRAP and fluorescence recovery of mCherry molecules conjugated to Tau. Marked squares represent the regions of bleaching immediately after photo-bleaching (0’–0 s after bleaching) and subsequent fluorescent recovery (88’–88 s after bleaching). **(B)** Obtained data of fluorescent recovery were normalized and recovery curves were built according to one-phase exponential association (see section “Materials and Methods”). **(C)** The graph represents the fitted data (by one-phase exponential association, see section “Materials and Methods”) of immobile fractions (collected on 88 s after photobleaching). Statistical analysis was performed by One-way ANOVA with Tukey HSD. Statistical significance is presented by ^∗^*P* < 0.05, ^∗∗^*P* < 0.01, ^∗∗∗^*P* < 0.001. Control, *n* = 82; Zn, *n* = 43; Zn + Ac-SKIP 10^–12^ M, *n* = 44; Zn + SKIP 10^–12^ M, *n* = 79; Zn + Ac-SKIP 10^–9^ M, *n* = 76; Zn + SKIP 10^–9^ M, *n* = 81.

In order to evaluate the protective activity of Ac-SKIP and SKIP against Tau-MT discharge we performed FRAP assay using zinc as Tau-MT dissociation agent (Craddock et al., 2012; Huang et al., 2014). Differentiated N1E-115 cells were transfected with a plasmid expressing mCherry-tagged Tau protein, and FRAP imaging (Figure 3A and Supplementary Figure S2) was performed after an hour of cell exposure to zinc alone or together with Ac-SKIP or SKIP (10^–12^ M, 10^–9^ M). MT regions decorated by mCherry-Tau were bleached (Figure 3A, marked squares, 0 s after bleaching) and recovery of mCherry fluorescence (Figure 3A, marked squares, 88 s after bleaching) resulted from interchange between MT-bound Tau (carrying bleached mCherry molecules) and previously unbound Tau proteins (carrying unbleached mCherry molecules). Thus, an unrecovered fraction of the initial mCherry fluorescence within a given bleached area indicated the immobile fraction mCherry-Tau proteins, reflecting Tau interaction with MTs. Subsequent analysis of the data obtained with a one-phase exponential association (Figures 3B,C) showed that zinc significantly abated Tau immobile fraction in comparison to the non-treated control (Figures 3B,C). Ac-SKIP and SKIP at both examined concentrations (10^–12^ M and 10^–9^ M) prevented excessive Tau release from MTs, induced by zinc, which was found statistically significant in comparison to zinc treatment alone (Figures 3B,C).

### Ac-SKIP and SKIP Prevent MT Disassembly, Induced by Zinc Intoxication

**FIGURE 4:**
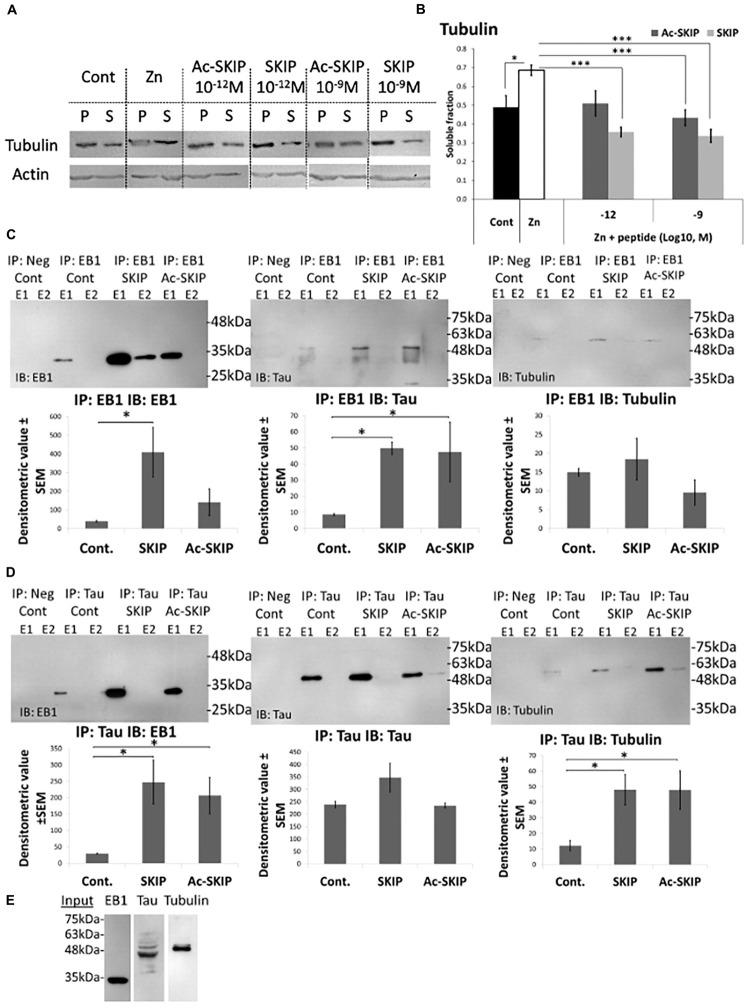
Ac-SKIP and SKIP effect on the polymerized vs. the soluble tubulin pool and the crosstalk between EB-Tau-tubulin. **(A)** Immunoblotting of polymerized (P) and soluble (S) protein fractions (obtained by polymerized vs. soluble tubulin assay, see section “Materials and Methods”) with tubulin antibodies. Cells were treated with zinc (400 μM, 4 h) with or without Ac-SKIP or SKIP (10^–12^ M and 10^–9^ M, 4 h), non-treated cells served as controls. **(B)** The graph represents the densitometric quantification of soluble tubulin ratios. The intensity of each band was quantified by densitometry and the soluble protein ratio was calculated by dividing the densitometric value of soluble proteins by the total protein content (S/[S + P]). Statistical analysis was performed by One Way ANOVA with Tukey HSD. ^∗^*P* < 0.05, ^∗∗^*P* < 0.01, ^∗∗∗^*P* < 0.001; Control, *n* = 15; Zn, *n* = 18; Zn + Ac-SKIP 10^–12^ M, *n* = 15; Zn + SKIP 10^–12^ M, *n* = 9; Zn + Ac-SKIP 10^–9^ M, *n* = 18; Zn + SKIP 10^–9^ M, *n* = 11. **(C,D)** A Co-IP assay was performed with EB1 or Tau antibodies, linked to agarose beads. SKIP (2 μg/sample) or Ac-SKIP (2 μg/sample), diluted into Pierce lysis buffer (see section “Materials and Methods”) or the equal volume of lysis buffer w/o peptides (IP: EB1 Cont; IP: Tau Cont) were added to cell lysate of differentiated SH-SY5Y cells, 15 min before EB1 or Tau column application (IP: EB1; IP: Tau). Sequential IP elution fractions (E1, E2) were further analyzed by immunoblotting with EB1, Tau, and Tubulin antibodies (IB: EB1; IB: Tau; IB: Tubulin). In addition, columns with free agarose beads were used as negative controls (IP: Neg cont). The intensity of each band was quantified by densitometry. The bar graph shows the ratio of band intensities (densitometric value ± SEM) obtained upon SKIP and Ac-SKIP treatments as compared with non-treated cells (Cont). Statistical analysis was performed by One Way ANOVA with LSD HSD. Experiments were independently repeated three times. ^∗^*P* < 0.05, ^∗∗^*P* < 0.01, ^∗∗∗^*P* < 0.001. **(E)** Cell lysates without column exposure were used as positive controls (Input). IP – immunoprecipitation, IB – immunoblot.

Further, we aimed to determine the protective effect of Ac-SKIP and SKIP against MT disassembly induced by zinc as an MT disruptor. We examined the relative levels of polymerized and soluble tubulin pools in differentiated N1E-115 cells, exposed to extracellular zinc (400 μM) alone or together with Ac-SKIP or SKIP (10^–12^ M and 10^–9^ M). After treatment cells were lysed (as described in section “Materials and Methods” section) and the tubulin levels of polymerized and soluble fractions were evaluated by western blotting (Figure 4A, Tubulin panel, and Supplementary Figure S3A) followed by densitometric quantification (Figure 4B). Non-treated control cells exhibited a nearly equal distribution of tubulin between soluble (S) and polymerized (P) fractions, while zinc treatment significantly increased the ratio of soluble to polymerized tubulin content (Figure 4A, Tubulin panel; Figure 4B). Ac-SKIP or SKIP added together with zinc exhibited reduced the tubulin content in the soluble fraction compared to “zinc” group, which was found statistically significant for all peptide treatments, except for Ac-SKIP at 10^–12^ M (Figure 4A, Tubulin panel; Figure 4B).

There was no observed effect on the actin-microfilament pool following neither zinc nor Ac-SKIP/SKIP treatment (Figure 4A, Actin panel, and Supplementary Figures S3B, S4), suggesting that zinc/Ac-SKIP/SKIP had selective effects on the MT cytoskeleton.

### SKIP/Ac-SKIP Enhance Tau-EB1 and Tau-Tubulin Interactions

To study the effect of SKIP and Ac-SKIP on the crosstalk between EB1-Tau and -tubulin, we performed co-immunoprecipitation (Co-IP) assays in differentiated human neuroblastoma SH-SY5Y cells as previously reported (Merenlender-Wagner et al., 2015; Ivashko-Pachima et al., 2017). We examined the effect of SKIP and Ac-SKIP on EB1-Tau interaction by protein complex immunoprecipitation (Co-IP) assays, using EB1 and Tau antibodies linked to agarose beads (Figure 4C, IP: EB1; Figure 4D, IP: Tau). The elution fractions, obtained from immunoprecipitations (IPs) performed with either anti-EB1 or anti-Tau, were analyzed by immunoblotting (IB) with EB1 and Tau antibodies (Figure 4C, IP: EB1 IB: Tau; Figure 4D, IP: Tau IB: EB1). Results showed that both SKIP and Ac-SKIP increased Tau-EB1 association, compared to the samples incubated without these peptides (Figure 4C, IP: EB1 Cont – control, IB: Tau; Figure 4D, IP: Tau Cont IB: EB1). IP: EB1 IB: EB1 showed a significant increase in EB1-EB1 interaction (EB1 homodimer formation) following SKIP treatment and moderate non-significant increase following Ac-SKIP, compared to the control (Figure 4C, IP: EB1 Cont IB: EB1). IP: Tau IB: Tau did not show significant differences in Tau-Tau association following incubation with either SKIP or Ac-SKIP (Figure 4D). Further immunoblotting analysis with tubulin antibodies suggested increased Tau-tubulin, interaction following treatments with SKIP and Ac-SKIP (Figure 4D, IP: Tau IB: tubulin), while EB1-tubulin association remained without significant changes (Figure 4C, IP: EB1 IB: tubulin). Cell lysates without column exposure were used as positive controls (Input, Figure 4E) and flow-through and washing fractions were also collected and analyzed by immunoblotting with EB1 and Tau antibodies (Supplementary Figure S5A, IP: EB1 IB: EB1; Supplementary Figure S5B, IP: Tau IB: Tau).

## Discussion

Maintenance of axonal structure and transport underlies neuronal signal transduction and connectivity, and provide a proper brain function (Rasband, 2010). It has been well-established that axonal degeneration (axonopathy) is a central pathogenic feature common to numerous human tauopathies, including AD (Kneynsberg et al., 2017). Atrophy of axon-reach white matter is significant in patients with mild cognitive impairment and extends widely in advanced AD cases, while the degree of atrophy is associated with loss of cognitive functioning (Huang and Auchus, 2007). Furthermore, immunohistochemical analyses of AD brains have revealed that formation of neuropil threads (Tau inclusions within dystrophic neurites) are a prominent AD neuropathological feature that appears before neurofibrillary tangles (NFTs) (Kowall and Kosik, 1987). Mechanistically, a clear correlation between Tau lesions and axonopathy has been demonstrated by multiple models, linking aberrant phosphoregulation of Tau to alterations in axonal transport, and deficits in axonal transport to dying-back degeneration of neurons (Kanaan et al., 2011, 2012, 2013).

The functional repertoire of Tau includes, among other characteristics, regulation of EB protein localization on MTs (Sayas et al., 2015). EBs are MT plus-end tracking proteins that decorate growing MT ends and recruit a variety of other proteins that connect MTs to various cellular structures (Jiang et al., 2012) and control MT dynamics (Komarova et al., 2009). EB-binding proteins directly interact with EBs through a core SxIP motif (Honnappa et al., 2009).

We have previously shown that EB1/3-targeting SKIP ameliorates impaired axonal transport and social memory deficits in the face of *Adnp* deficiency (Adnp^±^ mice) (Amram et al., 2016). Our current study focused on the molecular activity of SKIP and Ac-SKIP on the cellular compartment, relevant to the axonal transport – MTs, and MT-associated proteins Tau and EB1. We showed that SKIP and Ac-SKIP significantly increased the elongation and growth rate of MT plus-ends, mediated by EB1 proteins. Furthermore, the protective activity of SKIP and Ac-SKIP on Tau-MT interaction and MT integrity was assessed in the face of zinc intoxication. The well-established hypothesis of zinc dyshomeostasis in AD suggests aberrant zinc accumulation by Aβ-amyloid plaques, resulting in too low, or excessively high intracellular zinc concentrations (up to 1 mM) in zinc-enriched brain regions implicated in the cognitive functions and vulnerable to AD pathology (Deibel et al., 1996; Craddock et al., 2012; DeGrado et al., 2016). It has been suggested that high intracellular zinc concentrations (more than 100 μM) present adverse effects on nerve fibers during stimulation (Minami et al., 2006) and may lead to pathological implications and neuronal cell death (Frederickson et al., 2005; Sensi et al., 2009, 2011; Shuttleworth and Weiss, 2011). In addition, it has been shown that abnormally high concentrations (up to 250 μM) of zinc induce GSK-3β activation and Tau release from MTs (Boom et al., 2009). Neuronal loss has been observed when zinc levels reached 10–100 nM in the neuronal soma *in vitro* (Aizenman et al., 2000; Jiang et al., 2001; Bossy-Wetzel et al., 2004) and zinc influx of 300 μM causes ischemic neurodegeneration in rat brains and cortical cultures (Koh et al., 1996). It has also been found that NFTs and amyloid-beta (Aβ) plaques contain abnormally high levels of zinc (Bush et al., 1994a; Lee et al., 2018). Furthermore, it has been demonstrated that Aβ 1–40 (a major component of AD cerebral amyloid) specifically and in a saturable fashion binds zinc (Bush et al., 1994b) that could accelerate the Aβ plaques formation at 200 μM of zinc (Lee et al., 2018).

Here, we demonstrated an effect of SKIP and Ac-SKIP on the crosstalk between Tau, EB1, and MTs. In this respect, it has been previously shown that Tau directly interacts with EB proteins, modulating MT dynamics (Sayas et al., 2015). Based on the observed results, we suggest that the effect of SKIP and Ac-SKIP on MTs integrity and dynamics is mediated by increasing interaction between EB1 and Tau that is accompanied by the increased association of Tau with tubulin. In addition, SKIP and Ac-SKIP (the last with more moderate effect) enhance EB1 homodimer formation. EB proteins form homo- and heterodimers and play a master role in organizing dynamic protein networks in mammalian cells (Akhmanova and Steinmetz, 2015). EB1 homodimers have a higher affinity to the p150^glued^ – N-terminal CAP-Gly domain of the dynactin (Honnappa et al., 2006; Bjelic et al., 2012), which in turn acts as a co-factor for the MT motor protein dynein. EB1-dependent ordered recruitment of dynactin to the MT plus-end is required for efficient initiation of retrograde axonal transport (Moughamian et al., 2013). Furthermore, actin-MT linkers – spectraplakins promote axonal growth in an EB1-dependent manner and stabilize MTs in the face of pharmacologically induced depolymerization (Alves-Silva et al., 2012). Thus, increased EB1 homodimer formation by SKIP/Ac-SKIP may have a direct effect on axonal retrograde transport, axonal growth, and neuroprotection.

The differential potency and efficacy of SKIP and Ac-SKIP might be attributed to potential steric hindrance of the modified SKIP at low concentration, and differential residence time at higher concentrations affecting cellular survival. Regardless, since MT organization and dynamics is central in axonal MT cytoskeleton and transport our current study provided a compelling molecular explanation to the *in vivo* activity of SKIP, placing SKIP motif as a central focus for MT-based neuroprotection in tauopathies with axonal transport implication. As MT reduction in AD and aging is independent of Tau filament formation (Cash et al., 2003), our studies implicate a paucity/dysregulation of EB-interacting endogenous proteins, like ADNP (Oz et al., 2014; Malishkevich et al., 2016) as a contributing mechanism and further provides hope with SKIP-containing drug candidates in future *in vivo* studies.

## Data Availability Statement

All datasets generated for this study are included in the manuscript/Supplementary Files.

## Author Contributions

YI-P designed and performed the work and wrote the initial draft. IG inspired the project, guided the progress, provided all the required support, and finalized the manuscript.

## Conflict of Interest

IG serves as the Chief Scientific Officer of Coronis Neurosciences, developing CP102 (SKIP and Ac-SKIP) for neurological disorders. The remaining author declares that the research was conducted in the absence of any commercial or financial relationships that could be construed as a potential conflict of interest.
