# Corrigendum: A Novel Microtubule-Tau Association Enhancer and Neuroprotective Drug Candidate: Ac-SKIP

**DOI:** 10.3389/fncel.2021.687301

**Published:** 2021-05-26

**Authors:** Yanina Ivashko-Pachima, Illana Gozes

**Affiliations:** Dr. Diana and Zelman Elton (Elbaum) Laboratory for Molecular Neuroendocrinology, Department of Human Molecular Genetics and Biochemistry, Sackler Faculty of Medicine, Sagol School of Neuroscience, Adams Super Center for Brain Studies, Tel Aviv University, Tel Aviv, Israel

**Keywords:** tau, microtubules, EBs, ADNP, SKIP

In the original article, there were inconsistencies when comparing one by one the panels of Figure 4A in the main text and the representative extended immunoblot results in Supplementary Figure S3, as published. Thus, some of the representative images in the main text were unfortunately represented by extended mirrored, duplicated and rotated blots as explained below. It should be emphasized that for scientific rigor and data corroboration, each experiment was performed in triplicates and repeated three times and each immunoblot was exposed at least three times (three films), hence, the blot shown as a full image in the original supplementary material is a representative picture, as specifically stated in the original Supplementary Figure S3 legend. To maintain scientific rigor, there were 36 blots and almost 100 films. Our mistakes, corrected below, stem from an unfortunate mix-up coupled with an extremely high similarity between all repetitive experiments, we apologize for the mistakes.

**Figure 4:**
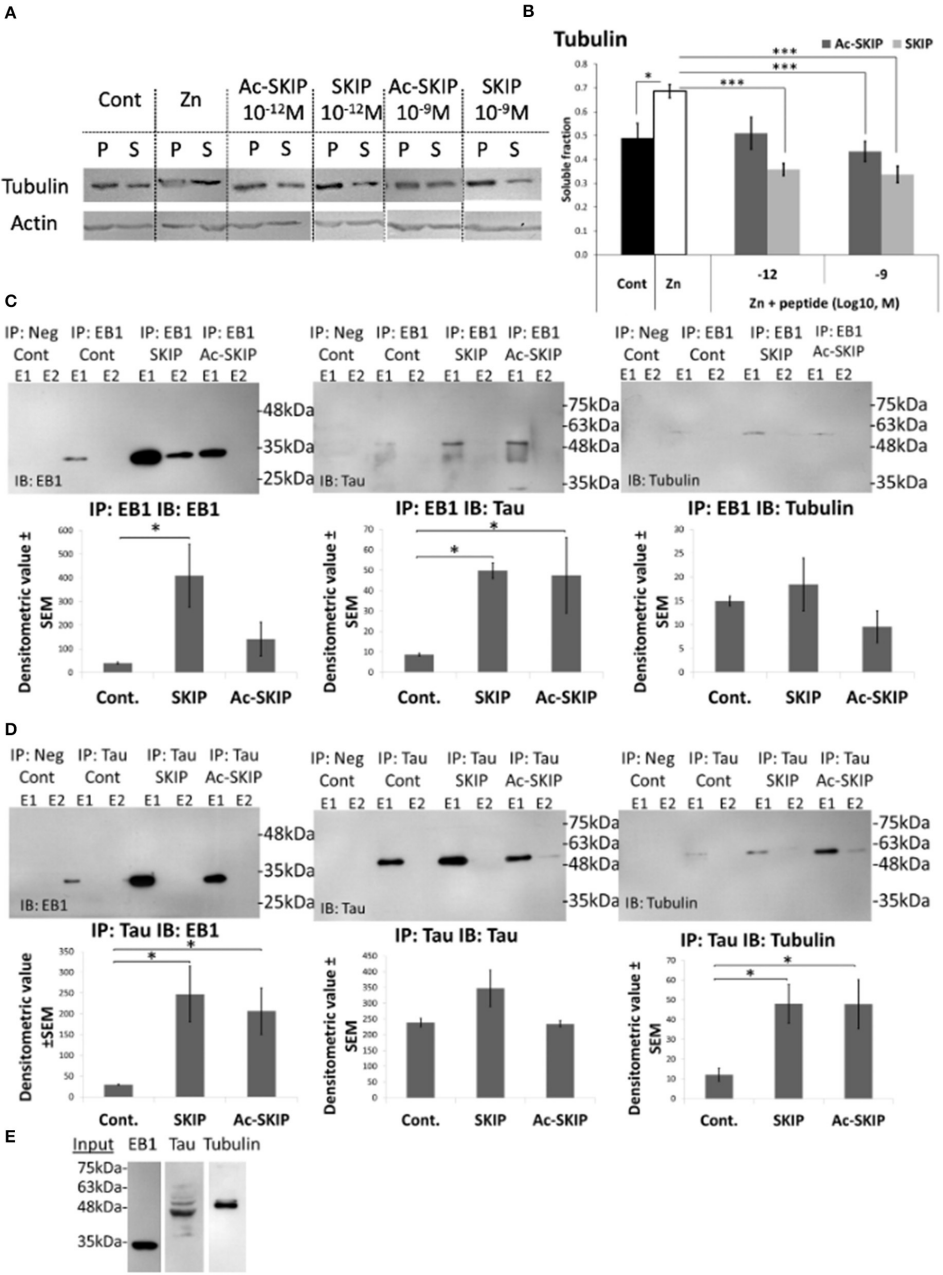
Ac-SKIP and SKIP effect on the polymerized vs. the soluble tubulin pool and the crosstalk between EB-Tau-tubulin. **(A)** Immunoblotting of polymerized (P) and soluble (S) protein fractions (obtained by polymerized vs. soluble tubulin assay, see section “Materials and Methods”) with tubulin antibodies. Cells were treated with zinc (400 μM, 4 h) with or without Ac-SKIP or SKIP (10^−12^ M and 10^−9^ M, 4 h), non-treated cells served as controls. **(B)** The graph represents the densitometric quantification of soluble tubulin ratios. The intensity of each band was quantified by densitometry and the soluble protein ratio was calculated by dividing the densitometric value of soluble proteins by the total protein content (S/[S + P]). Statistical analysis was performed by One Way ANOVA with Tukey HSD. ^*^P < 0.05, ^**^P < 0.01, ^***^P < 0.001; Control, *n* = 15; Zn, *n* = 18; Zn + Ac-SKIP 10^−12^ M, *n* = 15; Zn + SKIP 10^−12^ M, *n* = 9; Zn + Ac-SKIP 10^−9^ M, *n* = 18; Zn + SKIP 10^−9^ M, *n* = 11. **(C,D)** A Co-IP assay was performed with EB1 or Tau antibodies, linked to agarose beads. SKIP (2 μg/sample) or Ac-SKIP (2 μg/sample), diluted into Pierce lysis buffer (see section “Materials and Methods”) or the equal volume of lysis buffer w/o peptides (IP: EB1 Cont; IP: Tau Cont) were added to cell lysate of differentiated SH-SY5Y cells, 15 min before EB1 or Tau column application (IP: EB1; IP: Tau). Sequential IP elution fractions (E1, E2) were further analyzed by immunoblotting with EB1, Tau, and Tubulin antibodies (IB: EB1; IB: Tau; IB: Tubulin). In addition, columns with free agarose beads were used as negative controls (IP: Neg cont). The intensity of each band was quantified by densitometry. The bar graph shows the ratio of band intensities (densitometric value ± SEM) obtained upon SKIP and Ac-SKIP treatments as compared with non-treated cells (Cont). Statistical analysis was performed by One Way ANOVA with LSD HSD. Experiments were independently repeated three times. ^*^P < 0.05, ^**^P < 0.01, ^***^P < 0.001. **(E)** Cell lysates without column exposure were used as positive controls (Input). IP – immunoprecipitation, IB – immunoblot.

**Supplementary Figure S3:**
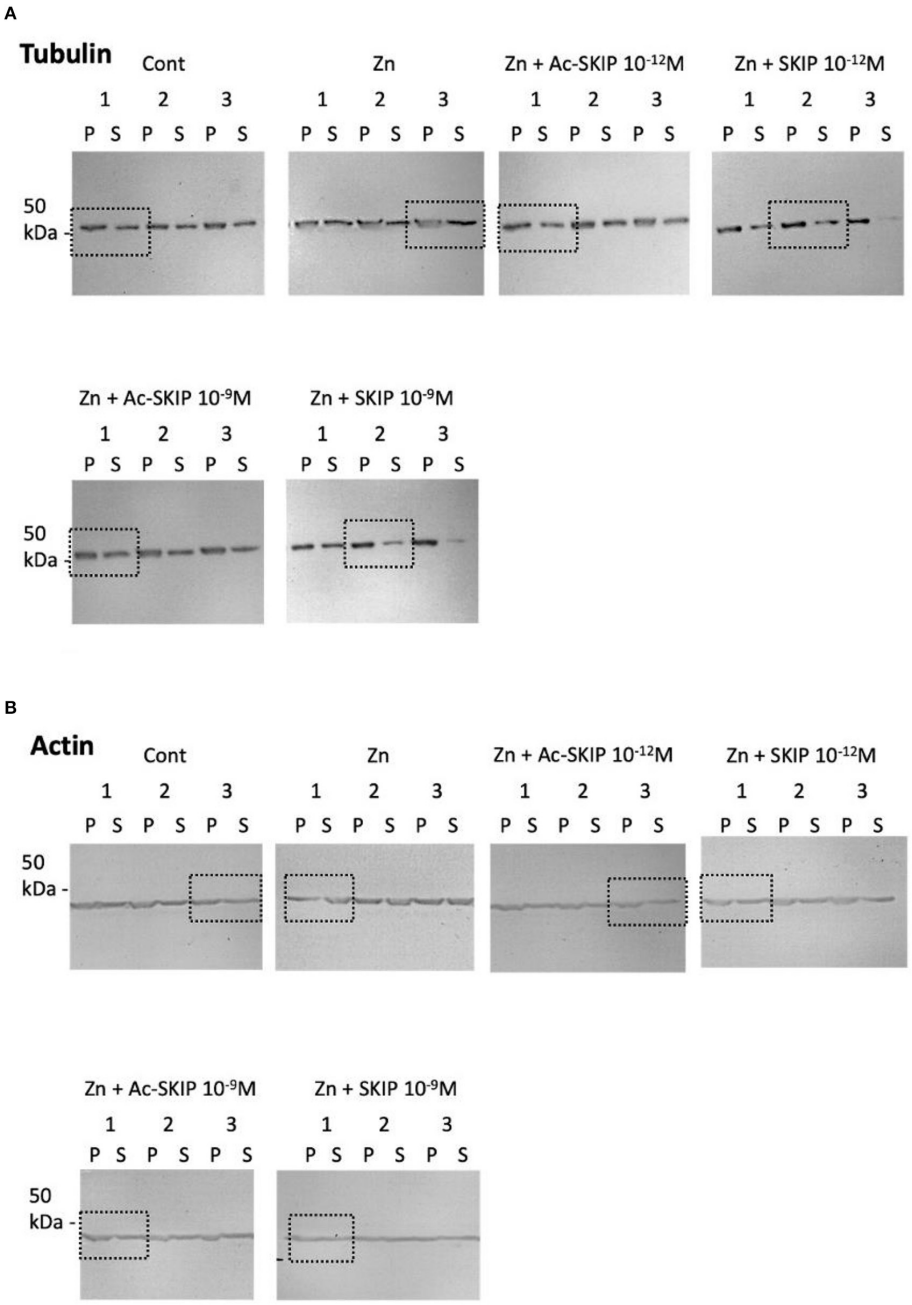
Representative pictures of immunoblot done with tubulin **(A)** and actin **(B)** antibodies. Polymerized (P) and soluble (S) tubulin pools were obtained from lysed differentiated N1E-115 cells without treatment (Control) or treated with zinc alone or together with Ac-SKIP or SKIP at 10^−12^M and 10^−9^M. Dotted squares represent areas, cut and displayed in the Figure 4 of the main text.

Corrections have been made to Figure 4, panel A, and Supplementary Figure S3. The corrected figures are shown below.

The authors apologize for these errors and state that they do not change the scientific conclusions of the article in any way. The original article has been updated.

