# Supplementary material for: A Novel Microtubule-Tau Association Enhancer and Neuroprotective Drug Candidate: Ac-SKIP
Source: Front Cell Neurosci. 2019 Oct 1;13:435. doi: 10.3389/fncel.2019.00435 (PMC6779860; doi:10.3389/fncel.2019.00435)
Supplement: Supplementary file 1 [file Data_Sheet_1.docx]

**Supplementary Material**


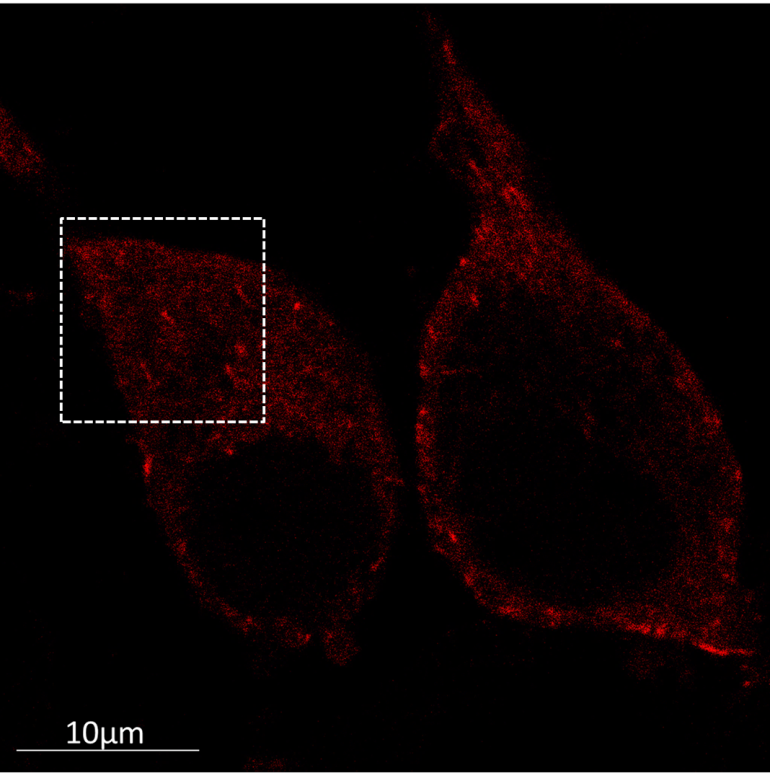

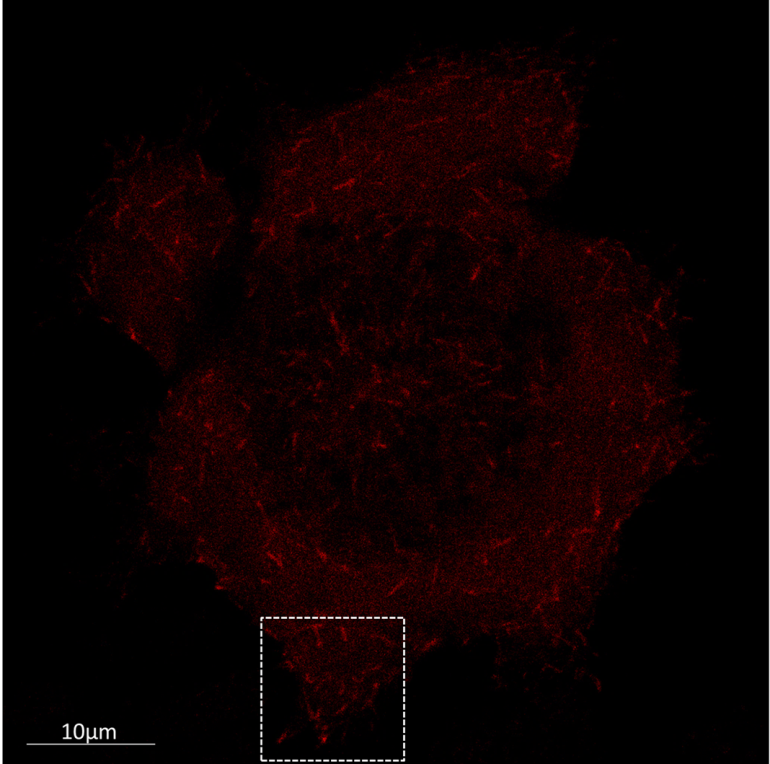

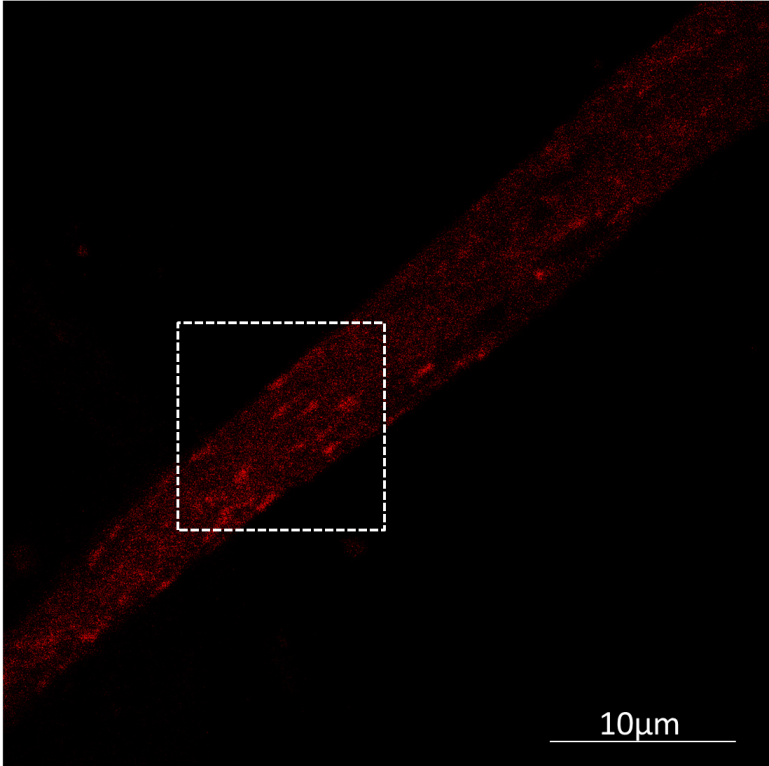


**C**

**B**

**A**

**SKIP 10^(-12)M**

**Ac-SKIP 10^(-12)M**

**Control**

**E**

**D**


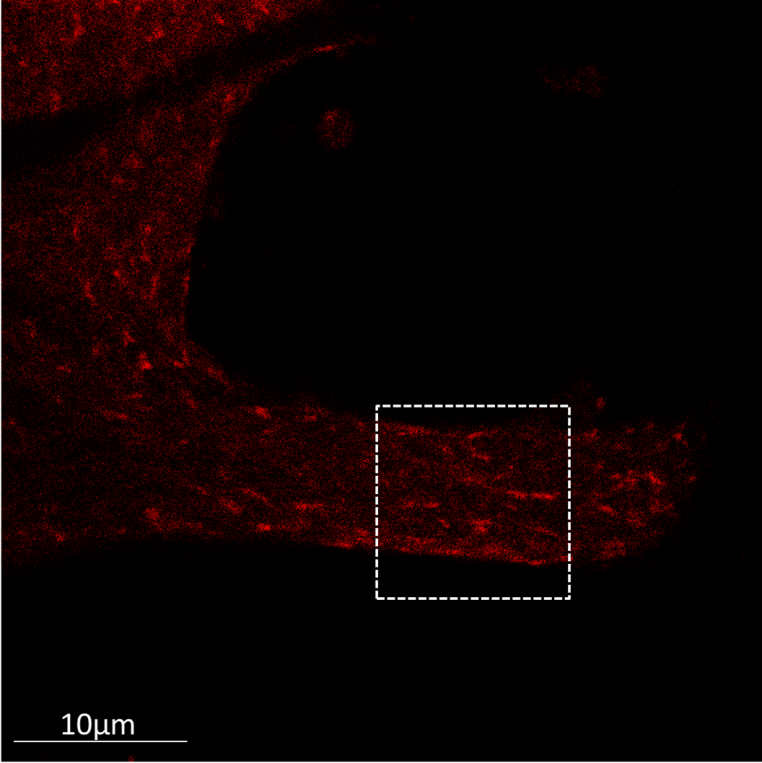

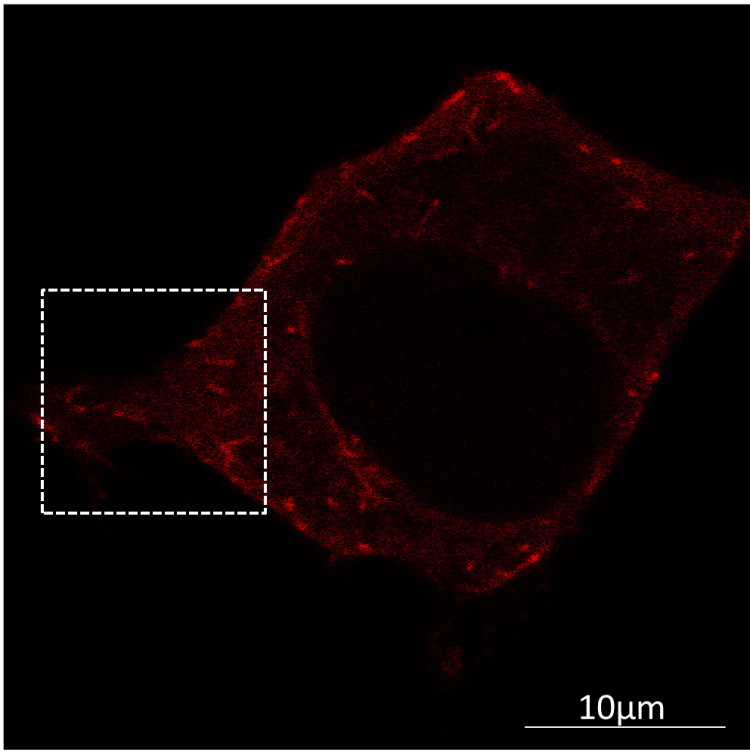


**SKIP 10^(-9)M**

**Ac-SKIP 10^(-9)M**

**Supplemental figure S1**: Representative pictures of live-imaging of differentiated N1E-115 cells expressing EB-RFP fusion protein. Cells without treatment were used as control **(A).**  The photos were taken 4hrs after treatment with Ac-SKIP 10^-12^M **(B),** SKIP 10^-12^M **(C),** Ac-SKIP 10^-9^M **(D)** and SKIP 10^-9^M **(E).** Dotted squares represent areas, cut and displayed in the figure 2 of the main text.


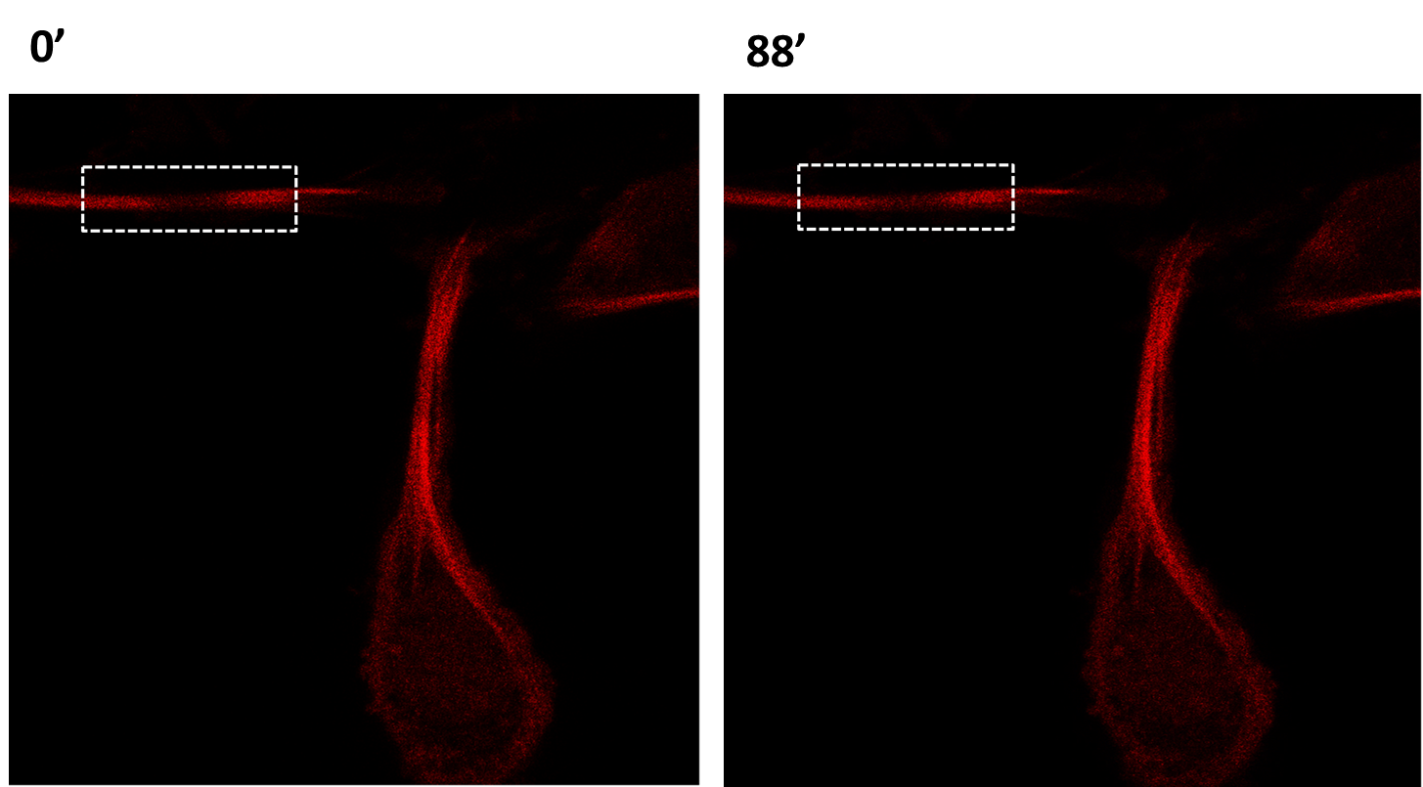

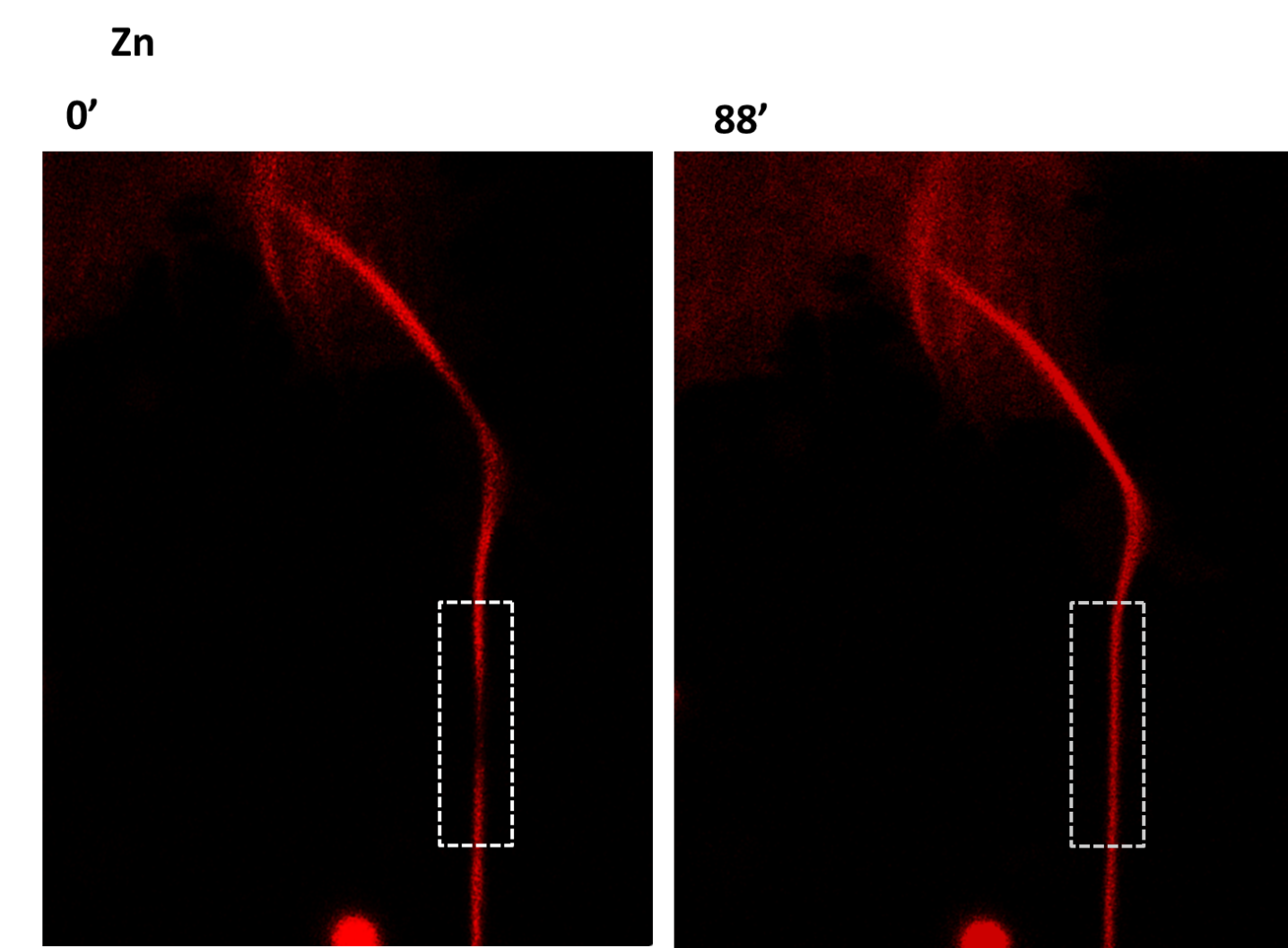


**B**

**A**

**Control**


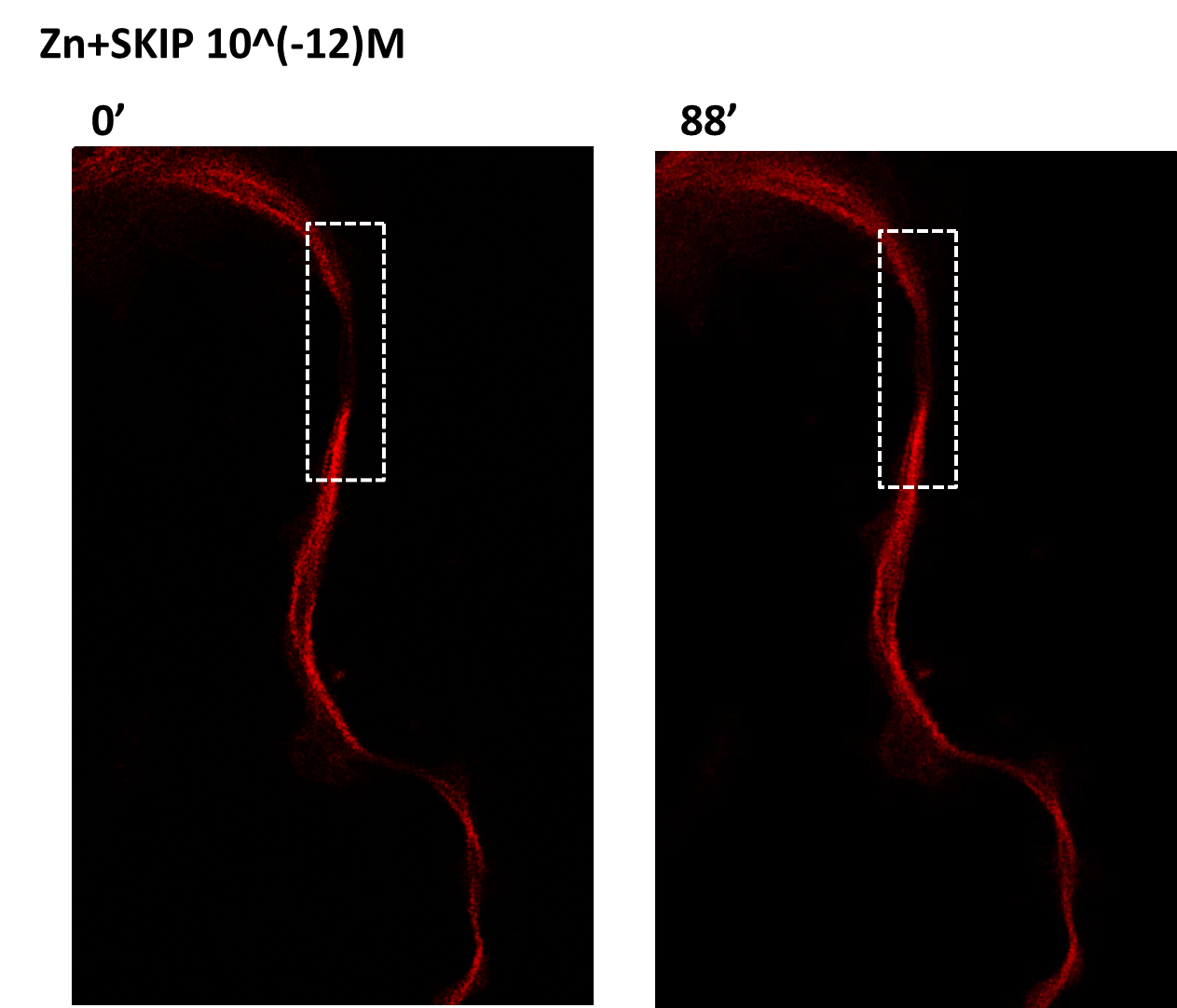

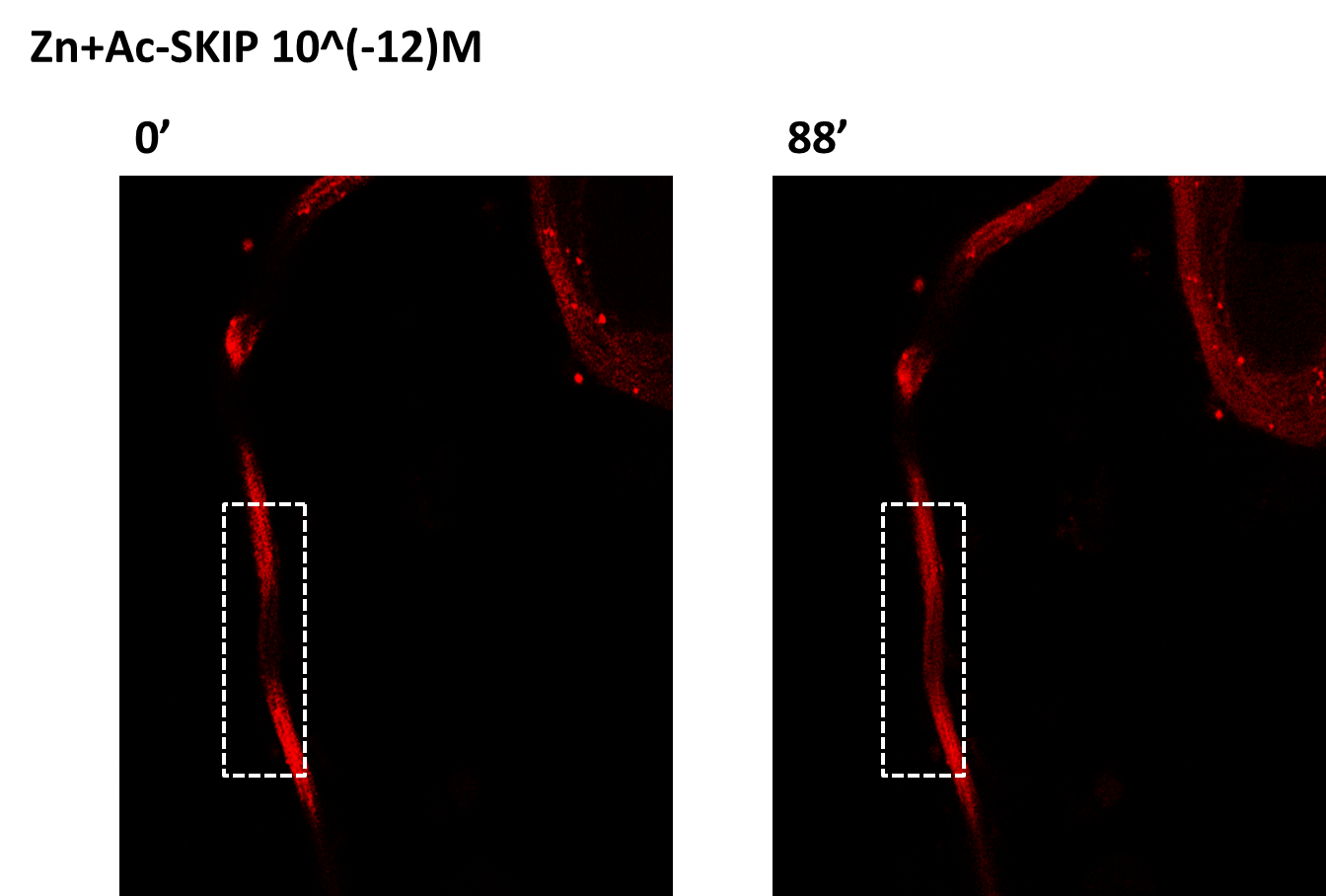


**D**

**C**


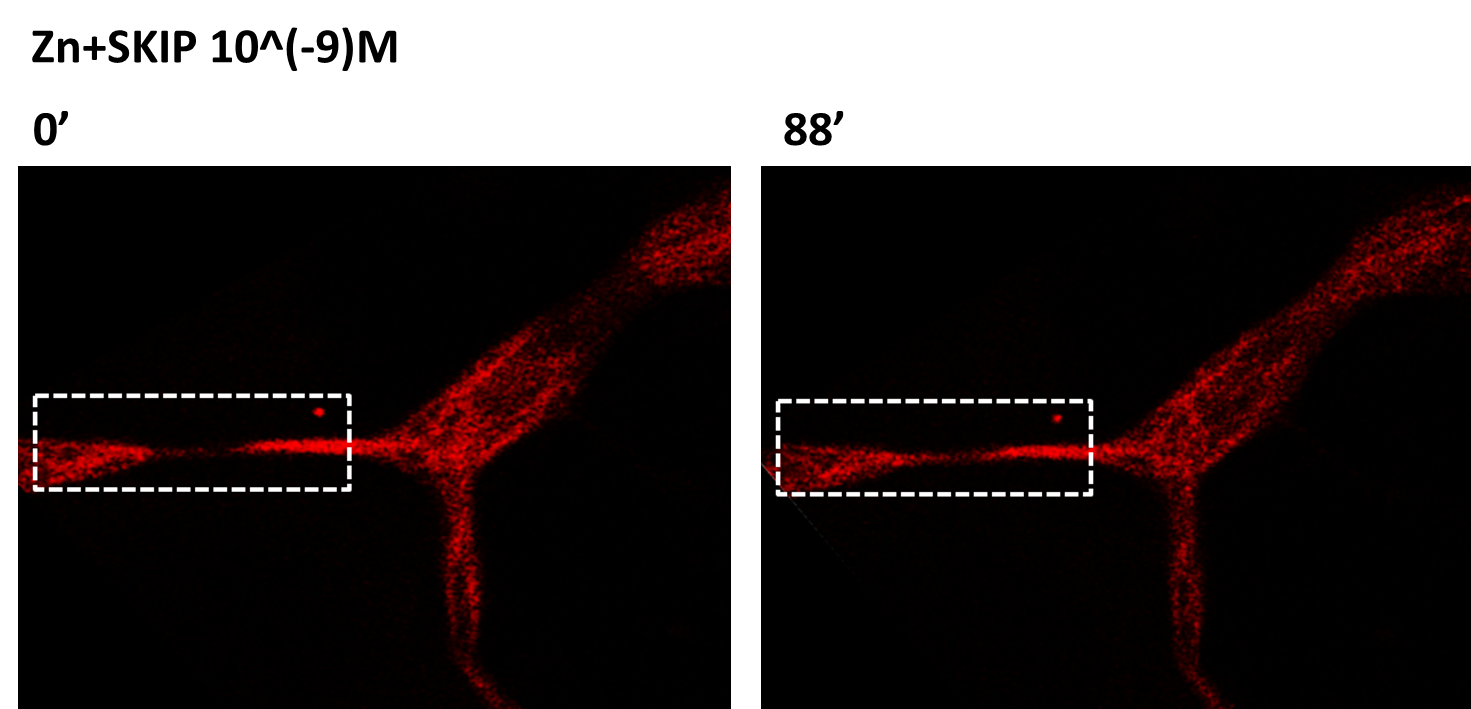

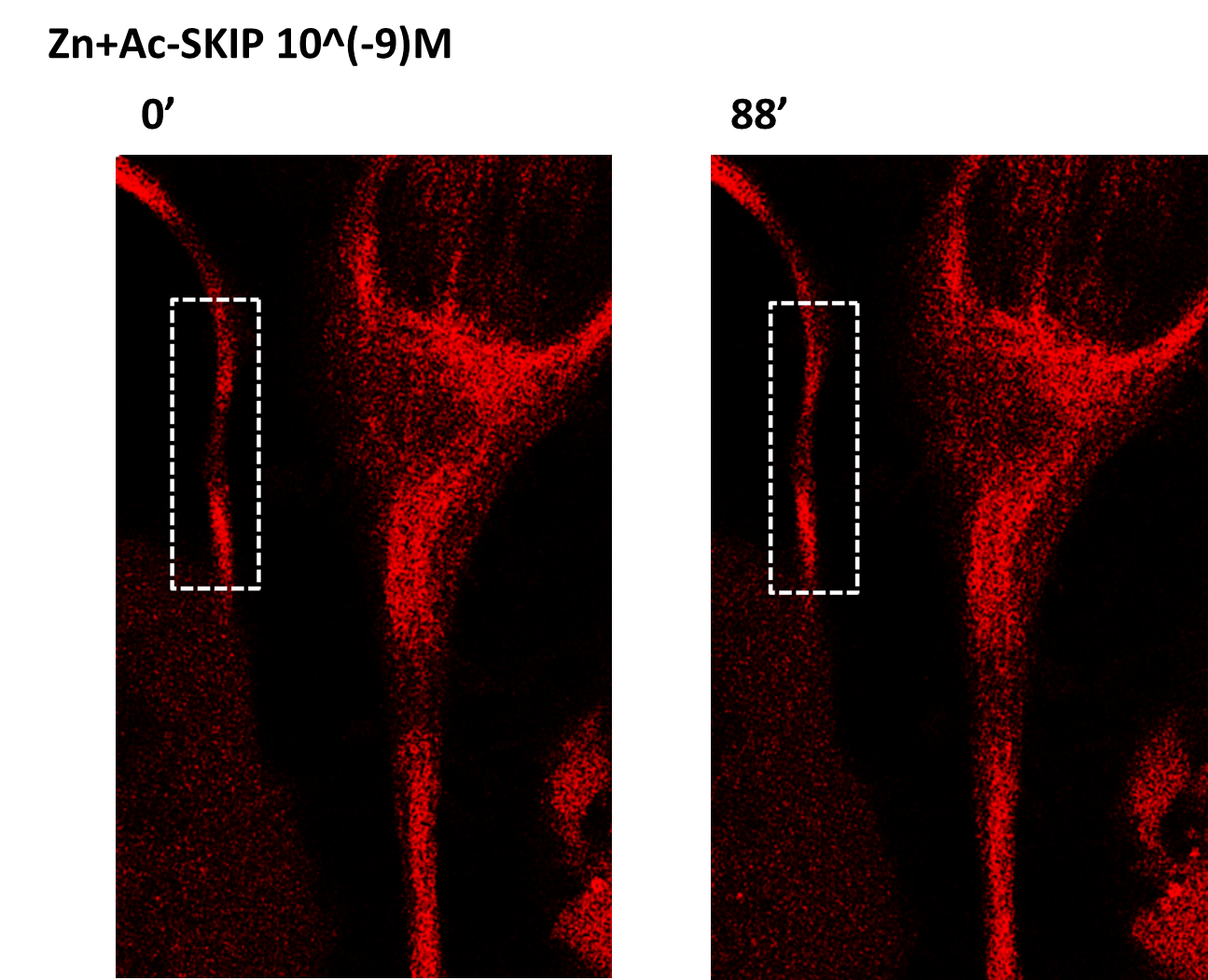


**E**

**F**

**Supplemental figure S2:** Representative images of fluorescence recovery after photobleaching (FRAP) assay performed on the differentiated N1E-115 cells, expressing mCherry-Tau fusion protein. Cells without treatment – “Control” **(A);** FRAP was done two hours after treatment with zinc alone **(B)** or together with Ac-SKIP 10^-12^M **(C),** SKIP 10^-12^M **(D),** Ac-SKIP 10^-9^M **(E),** and SKIP 10^-9^M **(F).** 0’ – 0 sec after bleaching; 88’ – 88 sec after bleaching. Dotted squares represent areas, cut and displayed in the figure 3 of the main text.


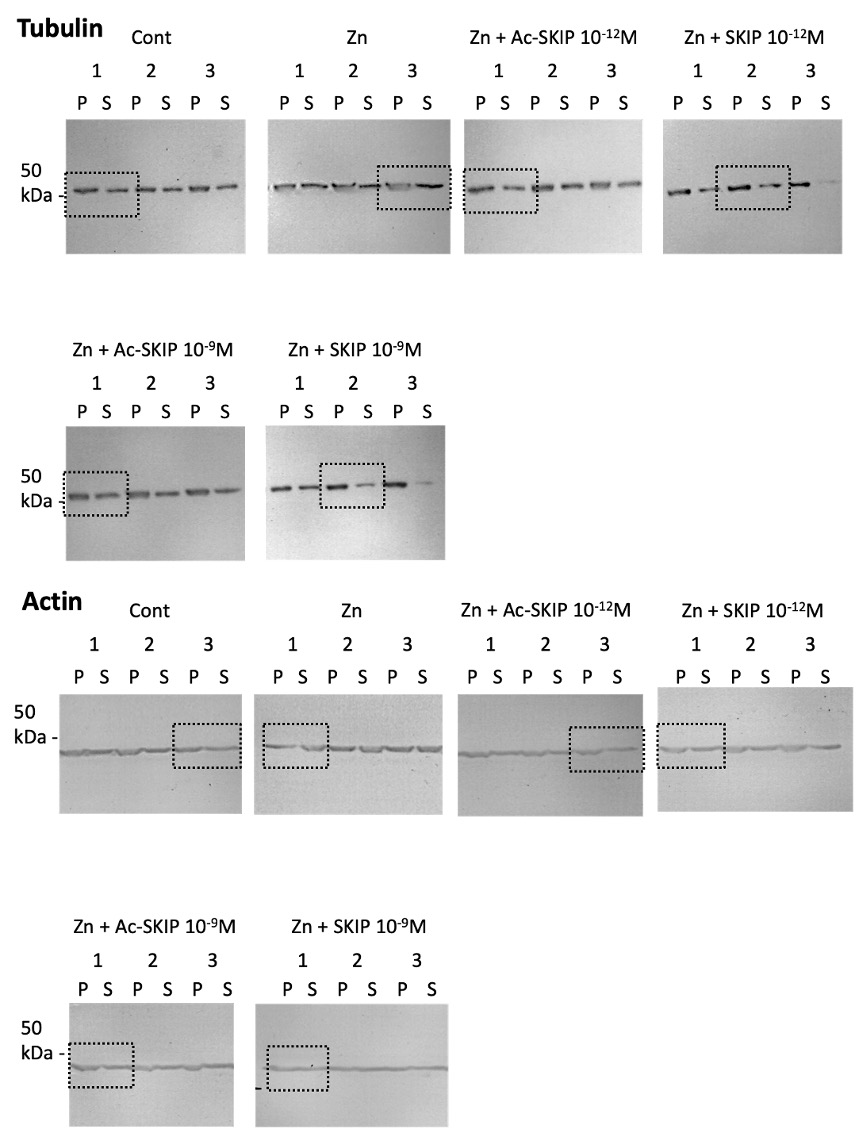


**A**

**B**


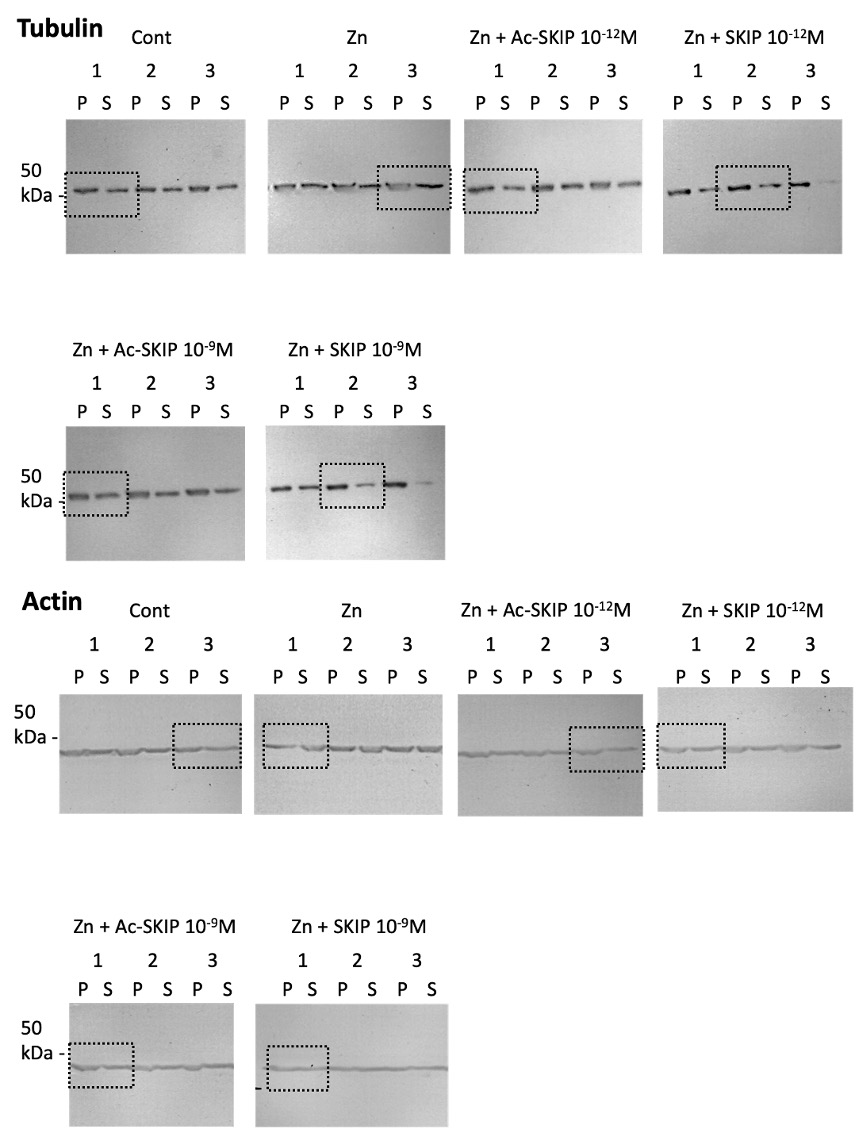


**Supplemental figure S3:** Representative pictures of immunoblot done with tubulin **(A)** and actin **(B)** antibodies. Polymerized (P) and soluble (S) tubulin pools were obtained from lysed differentiated N1E-115 cells without treatment (Control) or treated with zinc alone or together with Ac-SKIP or SKIP at 10^-12^M and 10^-9^M. Dotted squares represent areas, cut and displayed in the figure 4 of the main text.


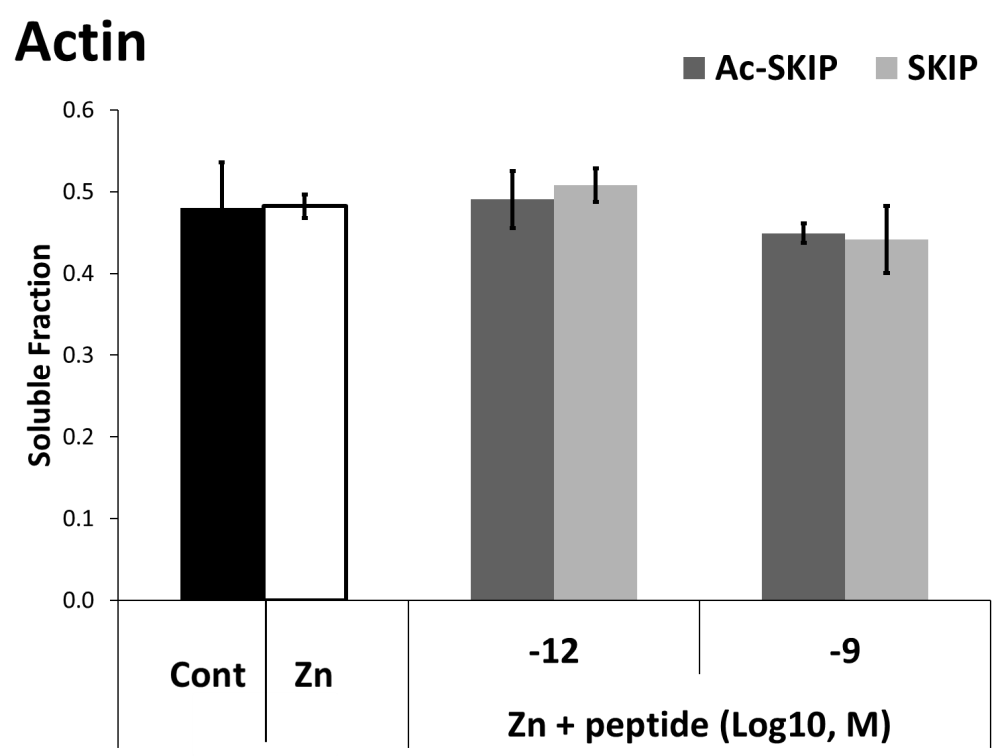


**Supplemental figure S4:** Graph represents the densitometric quantification of soluble actin ratios (Fig. 4A. main text). The intensity of each band was quantified by densitometry and the soluble ratio was calculated by dividing the densitometric value of soluble proteins by the total protein content (S/[S+P]). Statistical analysis was performed by One Way ANOVA with Tukey HSD.


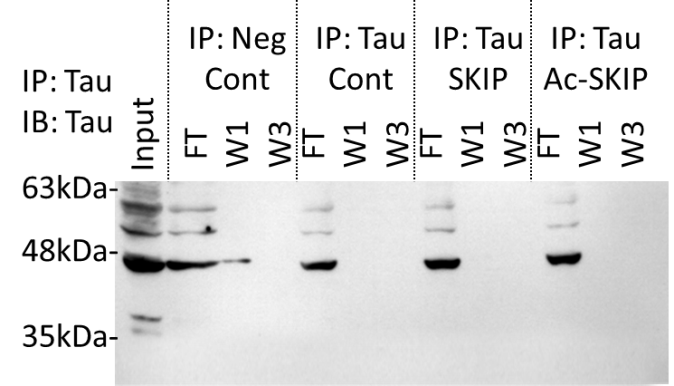

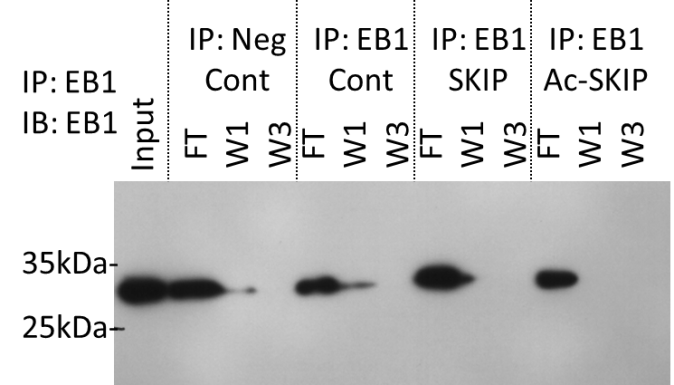


**A**

**B**

**Supplemental figure S5:** Immunoprecipitation (IP) was performed using EB1 (**A**, “IP: EB1”) and Tau (**B**,” IP: Tau”) antibodies with and without SKIP or Ac-SKIP (see “Materials and Methods”). IP without peptides was used as positive controls (“IP: EB1 Cont” and “IP: Tau Cont”), mock IP without antibodies – negative control (“IP: Neg Cont”). Input, flowthrough (FT), first and third washes (W1 and W3) fractions were collected and analyzed by immunoblot with EB1 (“IB: EB1”) and Tau (“IB: Tau”) antibodies.

**Statistical details of result displayed in the figure 1:** Statistical analysis was performed by One-way anova (SPSS 23) with Tukey HSD.

| **Descriptives** | | | | | | | | | | | | | | | | | | | | | | | | | | | | | |
| --- | --- | --- | --- | --- | --- | --- | --- | --- | --- | --- | --- | --- | --- | --- | --- | --- | --- | --- | --- | --- | --- | --- | --- | --- | --- | --- | --- | --- | --- |
| SKIP | | | | | | | | | | | | | | | | | | | | | | | | | | | | | |
|  | | | N | | | Mean | | | | Std. Deviation | | | | | Std. Error | | | | | 95% Confidence Interval for Mean | | | | | | Minimum | | Maximum | |
|  |  |  |  |  |  |  |  |  |  |  |  |  |  |  |  |  |  |  |  | Lower Bound | | | | Upper Bound | |  |  |  |  |
| Cont | | | 60 | | | .2721 | | | | .08392 | | | | | .00582 | | | | | .2802 | | | | .3032 | | .07 | | .67 | |
| Zn | | | 58 | | | .1330 | | | | .03529 | | | | | .00463 | | | | | .1066 | | | | .1251 | | .02 | | .20 | |
| Zn+SKIP-12 | | | 14 | | | .1918 | | | | .01464 | | | | | .00391 | | | | | .1833 | | | | .2002 | | .16 | | .21 | |
| Zn+SKIP-11 | | | 14 | | | .1939 | | | | .01396 | | | | | .00373 | | | | | .1859 | | | | .2020 | | .18 | | .23 | |
| Zn+SKIP-10 | | | 14 | | | .1852 | | | | .01252 | | | | | .00335 | | | | | .1779 | | | | .1924 | | .16 | | .20 | |
| Zn+SKIP-9 | | | 14 | | | .1913 | | | | .00789 | | | | | .00211 | | | | | .1867 | | | | .1958 | | .18 | | .21 | |
| Total | | | 322 | | | .2424 | | | | .09862 | | | | | .00550 | | | | | .2316 | | | | .2532 | | .02 | | .67 | |
| **ANOVA** | | | | | | | | | | | | | | | | | | | | | | |  |  |  |  |  |  |  |
| SKIP | | | | | | | | | | | | | | | | | | | | | | |  |  |  |  |  |  |  |
|  | | Sum of Squares | | | df | | | | Mean Square | | | | | F | | | | | Sig. | | | |  |  |  |  |  |  |  |
| Between Groups | | 1.585 | | | 5 | | | | .317 | | | | | 65.176 | | | | | .000 | | | |  |  |  |  |  |  |  |
| Within Groups | | 1.537 | | | 316 | | | | .005 | | | | |  | | | | |  | | | |  |  |  |  |  |  |  |
| Total | | 3.122 | | | 321 | | | |  | | | | |  | | | | |  | | | |  |  |  |  |  |  |  |
| **Multiple Comparisons** | | | | | | | | | | | | | | | | | | | | | | | | | |  |  |  |  |
| Dependent Variable: | | | | | | | | | | | | | | | | | | | | | | | | | |  |  |  |  |
| Tukey HSD | | | | | | | | | | | | | | | | | | | | | | | | | |  |  |  |  |
| (I) difSKIP | | | | | Mean Difference (I-J) | | | | Std. Error | | | | | Sig. | | | | | 95% Confidence Interval | | | | | | |  |  |  |  |
|  |  |  |  |  |  |  |  |  |  |  |  |  |  |  |  |  |  |  | Lower Bound | | | | Upper Bound | | |  |  |  |  |
| Cont | | Zn | | | .17582^*^ | | | | .01036 | | | | | .000 | | | | | .1461 | | | | .2055 | | |  |  |  |  |
|  |  | Zn+SKIP-12 | | | .09991^*^ | | | | .01926 | | | | | .000 | | | | | .0447 | | | | .1551 | | |  |  |  |  |
|  |  | Zn+SKIP-11 | | | .09777^*^ | | | | .01926 | | | | | .000 | | | | | .0426 | | | | .1530 | | |  |  |  |  |
|  |  | Zn+SKIP-10 | | | .10651^*^ | | | | .01926 | | | | | .000 | | | | | .0513 | | | | .1617 | | |  |  |  |  |
|  |  | Zn+SKIP-9 | | | .10042^*^ | | | | .01926 | | | | | .000 | | | | | .0452 | | | | .1556 | | |  |  |  |  |
| Zn | | Cont | | | -.17582^*^ | | | | .01036 | | | | | .000 | | | | | -.2055 | | | | -.1461 | | |  |  |  |  |
|  |  | Zn+SKIP-12 | | | -.07591^*^ | | | | .02077 | | | | | .004 | | | | | -.1355 | | | | -.0164 | | |  |  |  |  |
|  |  | Zn+SKIP-11 | | | -.07805^*^ | | | | .02077 | | | | | .003 | | | | | -.1376 | | | | -.0185 | | |  |  |  |  |
|  |  | Zn+SKIP-10 | | | -.06931^*^ | | | | .02077 | | | | | .012 | | | | | -.1289 | | | | -.0098 | | |  |  |  |  |
|  |  | Zn+SKIP-9 | | | -.07540^*^ | | | | .02077 | | | | | .004 | | | | | -.1350 | | | | -.0159 | | |  |  |  |  |
| Zn+SKIP-12 | | Cont | | | -.09991^*^ | | | | .01926 | | | | | .000 | | | | | -.1551 | | | | -.0447 | | |  |  |  |  |
|  |  | Zn | | | .07591^*^ | | | | .02077 | | | | | .004 | | | | | .0164 | | | | .1355 | | |  |  |  |  |
|  |  | Zn+SKIP-11 | | | -.00214 | | | | .02636 | | | | | 1.000 | | | | | -.0777 | | | | .0734 | | |  |  |  |  |
|  |  | Zn+SKIP-10 | | | .00660 | | | | .02636 | | | | | 1.000 | | | | | -.0690 | | | | .0822 | | |  |  |  |  |
|  |  | Zn+SKIP-9 | | | .00051 | | | | .02636 | | | | | 1.000 | | | | | -.0751 | | | | .0761 | | |  |  |  |  |
| Zn+SKIP-11 | | Cont | | | -.09777^*^ | | | | .01926 | | | | | .000 | | | | | -.1530 | | | | -.0426 | | |  |  |  |  |
|  |  | Zn | | | .07805^*^ | | | | .02077 | | | | | .003 | | | | | .0185 | | | | .1376 | | |  |  |  |  |
|  |  | Zn+SKIP-12 | | | .00214 | | | | .02636 | | | | | 1.000 | | | | | -.0734 | | | | .0777 | | |  |  |  |  |
|  |  | Zn+SKIP-10 | | | .00874 | | | | .02636 | | | | | .999 | | | | | -.0668 | | | | .0843 | | |  |  |  |  |
|  |  | Zn+SKIP-9 | | | .00265 | | | | .02636 | | | | | 1.000 | | | | | -.0729 | | | | .0782 | | |  |  |  |  |
| Zn+SKIP-10 | | Cont | | | -.10651^*^ | | | | .01926 | | | | | .000 | | | | | -.1617 | | | | -.0513 | | |  |  |  |  |
|  |  | Zn | | | .06931^*^ | | | | .02077 | | | | | .012 | | | | | .0098 | | | | .1289 | | |  |  |  |  |
|  |  | Zn+SKIP-12 | | | -.00660 | | | | .02636 | | | | | 1.000 | | | | | -.0822 | | | | .0690 | | |  |  |  |  |
|  |  | Zn+SKIP-11 | | | -.00874 | | | | .02636 | | | | | .999 | | | | | -.0843 | | | | .0668 | | |  |  |  |  |
|  |  | Zn+SKIP-9 | | | -.00609 | | | | .02636 | | | | | 1.000 | | | | | -.0817 | | | | .0695 | | |  |  |  |  |
| Zn+SKIP-9 | | Cont | | | -.10042^*^ | | | | .01926 | | | | | .000 | | | | | -.1556 | | | | -.0452 | | |  |  |  |  |
|  |  | Zn | | | .07540^*^ | | | | .02077 | | | | | .004 | | | | | .0159 | | | | .1350 | | |  |  |  |  |
|  |  | Zn+SKIP-12 | | | -.00051 | | | | .02636 | | | | | 1.000 | | | | | -.0761 | | | | .0751 | | |  |  |  |  |
|  |  | Zn+SKIP-11 | | | -.00265 | | | | .02636 | | | | | 1.000 | | | | | -.0782 | | | | .0729 | | |  |  |  |  |
|  |  | Zn+SKIP-10 | | | .00609 | | | | .02636 | | | | | 1.000 | | | | | -.0695 | | | | .0817 | | |  |  |  |  |
| *. The mean difference is significant at the 0.05 level. | | | | | | | | | | | | | | | | | | | | | | | | | |  |  |  |  |
| **Descriptives** | | | | | | | | | | | | | | | | | | | | | | | | | | | | |  |
| AcSKIP | | | | | | | | | | | | | | | | | | | | | | | | | | | | |  |
|  | N | | | Mean | | | | Std. Deviation | | | | | Std. Error | | | | | 95% Confidence Interval for Mean | | | | | | | Minimum | | Maximum | |  |
|  |  |  |  |  |  |  |  |  |  |  |  |  |  |  |  |  |  | Lower Bound | | | | Upper Bound | | |  |  |  |  |  |
| Cont | 60 | | | .2721 | | | | .08392 | | | | | .00582 | | | | | .2802 | | | | .3032 | | | .07 | | .67 | |  |
| Zn | 58 | | | .1330 | | | | .03529 | | | | | .00463 | | | | | .1066 | | | | .1251 | | | .02 | | .20 | |  |
| Zn+Ac-SKIP -12 | 22 | | | .1641 | | | | .03647 | | | | | .00778 | | | | | .1479 | | | | .1803 | | | .05 | | .23 | |  |
| Zn+Ac-SKIP -11 | 22 | | | .1864 | | | | .03736 | | | | | .00796 | | | | | .1698 | | | | .2029 | | | .09 | | .25 | |  |
| Zn+Ac-SKIP -10 | 22 | | | .2768 | | | | .07080 | | | | | .01510 | | | | | .2454 | | | | .3082 | | | .15 | | .38 | |  |
| Zn+Ac-SKIP -9 | 22 | | | .2709 | | | | .04471 | | | | | .00953 | | | | | .2511 | | | | .2907 | | | .17 | | .33 | |  |
| Total | 354 | | | .2462 | | | | .09829 | | | | | .00522 | | | | | .2359 | | | | .2565 | | | .02 | | .67 | |  |
| **ANOVA** | | | | | | | | | | | | | | | | | | | | | |  |  |  |  |  |  |  |  |
| AcSKIP | | | | | | | | | | | | | | | | | | | | | |  |  |  |  |  |  |  |  |
|  | Sum of Squares | | | df | | | Mean Square | | | | | F | | | | | Sig. | | | | |  |  |  |  |  |  |  |  |
| Between Groups | 1.677 | | | 5 | | | .335 | | | | | 67.324 | | | | | .000 | | | | |  |  |  |  |  |  |  |  |
| Within Groups | 1.733 | | | 348 | | | .005 | | | | |  | | | | |  | | | | |  |  |  |  |  |  |  |  |
| Total | 3.410 | | | 353 | | |  | | | | |  | | | | |  | | | | |  |  |  |  |  |  |  |  |
| **Multiple Comparisons** | | | | | | | | | | | | | | | | | | | | | | | | | |  |  |  |  |
| Dependent Variable: | | | | | | | | | | | | | | | | | | | | | | | | | |  |  |  |  |
| Tukey HSD | | | | | | | | | | | | | | | | | | | | | | | | | |  |  |  |  |
| (I) difff | | | | Mean Difference (I-J) | | | Std. Error | | | | Sig. | | | | | 95% Confidence Interval | | | | | | | | | |  |  |  |  |
|  |  |  |  |  |  |  |  |  |  |  |  |  |  |  |  | Lower Bound | | | | | Upper Bound | | | | |  |  |  |  |
| Cont | Zn | | | .17582^*^ | | | .01048 | | | | .000 | | | | | .1458 | | | | | .2059 | | | | |  |  |  |  |
|  | Zn+Ac-SKIP -12 | | | .12759^*^ | | | .01582 | | | | .000 | | | | | .0822 | | | | | .1729 | | | | |  |  |  |  |
|  | Zn+Ac-SKIP -11 | | | .10532^*^ | | | .01582 | | | | .000 | | | | | .0600 | | | | | .1507 | | | | |  |  |  |  |
|  | Zn+Ac-SKIP -10 | | | .01486 | | | .01582 | | | | .936 | | | | | -.0305 | | | | | .0602 | | | | |  |  |  |  |
|  | Zn+Ac-SKIP -9 | | | .02077 | | | .01582 | | | | .778 | | | | | -.0246 | | | | | .0661 | | | | |  |  |  |  |
| Zn | Cont | | | -.17582^*^ | | | .01048 | | | | .000 | | | | | -.2059 | | | | | -.1458 | | | | |  |  |  |  |
|  | Zn+Ac-SKIP -12 | | | -.04823 | | | .01767 | | | | .072 | | | | | -.0989 | | | | | .0024 | | | | |  |  |  |  |
|  | Zn+Ac-SKIP -11 | | | -.07050^*^ | | | .01767 | | | | .001 | | | | | -.1211 | | | | | -.0199 | | | | |  |  |  |  |
|  | Zn+Ac-SKIP -10 | | | -.16096^*^ | | | .01767 | | | | .000 | | | | | -.2116 | | | | | -.1103 | | | | |  |  |  |  |
|  | Zn+Ac-SKIP -9 | | | -.15505^*^ | | | .01767 | | | | .000 | | | | | -.2057 | | | | | -.1044 | | | | |  |  |  |  |
| Zn+Ac-SKIP -12 | Cont | | | -.12759^*^ | | | .01582 | | | | .000 | | | | | -.1729 | | | | | -.0822 | | | | |  |  |  |  |
|  | Zn | | | .04823 | | | .01767 | | | | .072 | | | | | -.0024 | | | | | .0989 | | | | |  |  |  |  |
|  | Zn+Ac-SKIP -11 | | | -.02227 | | | .02128 | | | | .902 | | | | | -.0833 | | | | | .0387 | | | | |  |  |  |  |
|  | Zn+Ac-SKIP -10 | | | -.11273^*^ | | | .02128 | | | | .000 | | | | | -.1737 | | | | | -.0517 | | | | |  |  |  |  |
|  | Zn+Ac-SKIP -9 | | | -.10682^*^ | | | .02128 | | | | .000 | | | | | -.1678 | | | | | -.0458 | | | | |  |  |  |  |
| Zn+Ac-SKIP -11 | Cont | | | -.10532^*^ | | | .01582 | | | | .000 | | | | | -.1507 | | | | | -.0600 | | | | |  |  |  |  |
|  | Zn | | | .07050^*^ | | | .01767 | | | | .001 | | | | | .0199 | | | | | .1211 | | | | |  |  |  |  |
|  | Zn+Ac-SKIP -12 | | | .02227 | | | .02128 | | | | .902 | | | | | -.0387 | | | | | .0833 | | | | |  |  |  |  |
|  | Zn+Ac-SKIP -10 | | | -.09045^*^ | | | .02128 | | | | .000 | | | | | -.1514 | | | | | -.0295 | | | | |  |  |  |  |
|  | Zn+Ac-SKIP -9 | | | -.08455^*^ | | | .02128 | | | | .001 | | | | | -.1455 | | | | | -.0236 | | | | |  |  |  |  |
| Zn+Ac-SKIP -10 | Cont | | | -.01486 | | | .01582 | | | | .936 | | | | | -.0602 | | | | | .0305 | | | | |  |  |  |  |
|  | Zn | | | .16096^*^ | | | .01767 | | | | .000 | | | | | .1103 | | | | | .2116 | | | | |  |  |  |  |
|  | Zn+Ac-SKIP -12 | | | .11273^*^ | | | .02128 | | | | .000 | | | | | .0517 | | | | | .1737 | | | | |  |  |  |  |
|  | Zn+Ac-SKIP -11 | | | .09045^*^ | | | .02128 | | | | .000 | | | | | .0295 | | | | | .1514 | | | | |  |  |  |  |
|  | Zn+Ac-SKIP -9 | | | .00591 | | | .02128 | | | | 1.000 | | | | | -.0551 | | | | | .0669 | | | | |  |  |  |  |
| Zn+Ac-SKIP -9 | Cont | | | -.02077 | | | .01582 | | | | .778 | | | | | -.0661 | | | | | .0246 | | | | |  |  |  |  |
|  | Zn | | | .15505^*^ | | | .01767 | | | | .000 | | | | | .1044 | | | | | .2057 | | | | |  |  |  |  |
|  | Zn+Ac-SKIP -12 | | | .10682^*^ | | | .02128 | | | | .000 | | | | | .0458 | | | | | .1678 | | | | |  |  |  |  |
|  | Zn+Ac-SKIP -11 | | | .08455^*^ | | | .02128 | | | | .001 | | | | | .0236 | | | | | .1455 | | | | |  |  |  |  |
|  | Zn+Ac-SKIP -10 | | | -.00591 | | | .02128 | | | | 1.000 | | | | | -.0669 | | | | | .0551 | | | | |  |  |  |  |
| *. The mean difference is significant at the 0.05 level. | | | | | | | | | | | | | | | | | | | | | | | | | |  |  |  |  |

**Statistical details of result displayed in the figure 2:** Statistical analysis was performed by One-way anova (SPSS 23) with Tukey HSD.

| **Descriptives** | | | | | | | | | |
| --- | --- | --- | --- | --- | --- | --- | --- | --- | --- |
|  | | N | Mean | Std. Deviation | Std. Error | 95% Confidence Interval for Mean | | Minimum | Maximum |
|  |  |  |  |  |  | Lower Bound | Upper Bound |  |  |
| TrackLength | Contrl | 47 | 1.1866 | .31426 | .04584 | 1.0943 | 1.2789 | .69 | 1.98 |
|  | Ac-SKIP -12 | 30 | 1.3707 | .34036 | .06214 | 1.2436 | 1.4978 | .86 | 2.01 |
|  | SKIP -12 | 20 | 1.0865 | .18505 | .04138 | .9999 | 1.1731 | .77 | 1.46 |
|  | Ac-SKIP -9 | 65 | 1.5688 | .39478 | .04897 | 1.4710 | 1.6667 | .73 | 2.51 |
|  | SKIP -9 | 43 | 1.6908 | .51278 | .07820 | 1.5330 | 1.8486 | .93 | 2.61 |
|  | Total | 205 | 1.4307 | .43723 | .03054 | 1.3705 | 1.4909 | .69 | 2.61 |
| CometSpeed | Contrl | 47 | .1221 | .03188 | .00465 | .1127 | .1314 | .08 | .19 |
|  | Ac-SKIP -12 | 30 | .1121 | .01769 | .00323 | .1055 | .1187 | .06 | .15 |
|  | SKIP -12 | 20 | .1202 | .01082 | .00242 | .1151 | .1253 | .10 | .14 |
|  | Ac-SKIP -9 | 64 | .1447 | .02335 | .00292 | .1388 | .1505 | .11 | .21 |
|  | SKIP -9 | 43 | .1463 | .02004 | .00306 | .1401 | .1525 | .11 | .19 |
|  | Total | 204 | .1326 | .02696 | .00189 | .1289 | .1363 | .06 | .21 |

| **ANOVA** | | | | | | |
| --- | --- | --- | --- | --- | --- | --- |
|  | | Sum of Squares | df | Mean Square | F | Sig. |
| TrackLength | Between Groups | 9.427 | 4 | 2.357 | 15.939 | .000 |
|  | Within Groups | 29.571 | 200 | .148 |  |  |
|  | Total | 38.998 | 204 |  |  |  |
| CometSpeed | Between Groups | .038 | 4 | .010 | 17.416 | .000 |
|  | Within Groups | .109 | 199 | .001 |  |  |
|  | Total | .148 | 203 |  |  |  |

| **Multiple Comparisons** | | | | | | | | | | | | |
| --- | --- | --- | --- | --- | --- | --- | --- | --- | --- | --- | --- | --- |
| Tukey HSD | | | | | | | | | | | | |
| Dependent Variable | | | | | Mean Difference (I-J) | | Std. Error | | Sig. | | 95% Confidence Interval | |
|  |  |  |  |  |  |  |  |  |  |  | Lower Bound | Upper Bound |
| TrackLength | | Contrl | | Ac-SKIP -12 | -.18407 | | .08986 | | .247 | | -.4314 | .0633 |
|  |  |  |  | SKIP -12 | .10010 | | .10266 | | .866 | | -.1825 | .3827 |
|  |  |  |  | Ac-SKIP -9 | -.38224^*^ | | .07362 | | .000 | | -.5849 | -.1796 |
|  |  |  |  | SKIP -9 | -.50417^*^ | | .08114 | | .000 | | -.7275 | -.2808 |
|  |  | Ac-SKIP -12 | | Contrl | .18407 | | .08986 | | .247 | | -.0633 | .4314 |
|  |  |  |  | SKIP -12 | .28417 | | .11100 | | .082 | | -.0214 | .5897 |
|  |  |  |  | Ac-SKIP -9 | -.19817 | | .08487 | | .138 | | -.4318 | .0355 |
|  |  |  |  | SKIP -9 | -.32009^*^ | | .09147 | | .005 | | -.5719 | -.0683 |
|  |  | SKIP -12 | | Contrl | -.10010 | | .10266 | | .866 | | -.3827 | .1825 |
|  |  |  |  | Ac-SKIP -12 | -.28417 | | .11100 | | .082 | | -.5897 | .0214 |
|  |  |  |  | Ac-SKIP -9 | -.48234^*^ | | .09832 | | .000 | | -.7530 | -.2117 |
|  |  |  |  | SKIP -9 | -.60426^*^ | | .10407 | | .000 | | -.8908 | -.3178 |
|  |  | Ac-SKIP -9 | | Contrl | .38224^*^ | | .07362 | | .000 | | .1796 | .5849 |
|  |  |  |  | Ac-SKIP -12 | .19817 | | .08487 | | .138 | | -.0355 | .4318 |
|  |  |  |  | SKIP -12 | .48234^*^ | | .09832 | | .000 | | .2117 | .7530 |
|  |  |  |  | SKIP -9 | -.12192 | | .07559 | | .491 | | -.3300 | .0862 |
|  |  | SKIP -9 | | Contrl | .50417^*^ | | .08114 | | .000 | | .2808 | .7275 |
|  |  |  |  | Ac-SKIP -12 | .32009^*^ | | .09147 | | .005 | | .0683 | .5719 |
|  |  |  |  | SKIP -12 | .60426^*^ | | .10407 | | .000 | | .3178 | .8908 |
|  |  |  |  | Ac-SKIP -9 | .12192 | | .07559 | | .491 | | -.0862 | .3300 |
| CometSpeed | | Contrl | | Ac-SKIP -12 | .00994 | | .00548 | | .368 | | -.0051 | .0250 |
|  |  |  |  | SKIP -12 | .00186 | | .00626 | | .998 | | -.0154 | .0191 |
|  |  |  |  | Ac-SKIP -9 | -.02260^*^ | | .00450 | | .000 | | -.0350 | -.0102 |
|  |  |  |  | SKIP -9 | -.02424^*^ | | .00494 | | .000 | | -.0379 | -.0106 |
|  |  | Ac-SKIP -12 | | Contrl | -.00994 | | .00548 | | .368 | | -.0250 | .0051 |
|  |  |  |  | SKIP -12 | -.00808 | | .00676 | | .754 | | -.0267 | .0105 |
|  |  |  |  | Ac-SKIP -9 | -.03254^*^ | | .00518 | | .000 | | -.0468 | -.0183 |
|  |  |  |  | SKIP -9 | -.03418^*^ | | .00557 | | .000 | | -.0495 | -.0188 |
|  |  | SKIP -12 | | Contrl | -.00186 | | .00626 | | .998 | | -.0191 | .0154 |
|  |  |  |  | Ac-SKIP -12 | .00808 | | .00676 | | .754 | | -.0105 | .0267 |
|  |  |  |  | Ac-SKIP -9 | -.02445^*^ | | .00600 | | .001 | | -.0410 | -.0079 |
|  |  |  |  | SKIP -9 | -.02610^*^ | | .00634 | | .001 | | -.0436 | -.0086 |
|  |  | Ac-SKIP -9 | | Contrl | .02260^*^ | | .00450 | | .000 | | .0102 | .0350 |
|  |  |  |  | Ac-SKIP -12 | .03254^*^ | | .00518 | | .000 | | .0183 | .0468 |
|  |  |  |  | SKIP -12 | .02445^*^ | | .00600 | | .001 | | .0079 | .0410 |
|  |  |  |  | SKIP -9 | -.00164 | | .00462 | | .997 | | -.0144 | .0111 |
|  |  | SKIP -9 | | Contrl | .02424^*^ | | .00494 | | .000 | | .0106 | .0379 |
|  |  |  |  | Ac-SKIP -12 | .03418^*^ | | .00557 | | .000 | | .0188 | .0495 |
|  |  |  |  | SKIP -12 | .02610^*^ | | .00634 | | .001 | | .0086 | .0436 |
|  |  |  |  | Ac-SKIP -9 | .00164 | | .00462 | | .997 | | -.0111 | .0144 |
| *. The mean difference is significant at the 0.05 level. | | | | | | | | | | | | |
| **Track Length** | | | | | | | | | | | | |
| Tukey HSD^a,b^ | | | | | | | | | | | | |
| diff | N | | Subset for alpha = 0.05 | | | | | | |  |  |  |
|  |  |  | 1 | | 2 | 3 | | 4 | |  |  |  |
| SKIP -12 | 20 | | 1.0865 | |  |  | |  | |  |  |  |
| Contrl | 47 | | 1.1866 | | 1.1866 |  | |  | |  |  |  |
| Ac-SKIP -12 | 30 | |  | | 1.3707 | 1.3707 | |  | |  |  |  |
| Ac-SKIP -9 | 65 | |  | |  | 1.5688 | | 1.5688 | |  |  |  |
| SKIP -9 | 43 | |  | |  |  | | 1.6908 | |  |  |  |
| Sig. |  | | .813 | | .270 | .202 | | .676 | |  |  |  |
| Means for groups in homogeneous subsets are displayed. | | | | | | | | | |  |  |  |
| a. Uses Harmonic Mean Sample Size = 34.904. | | | | | | | | | |  |  |  |
| b. The group sizes are unequal. The harmonic mean of the group sizes is used. Type I error levels are not guaranteed. | | | | | | | | | |  |  |  |

| **CometSpeed** | | | |
| --- | --- | --- | --- |
| Tukey HSD^a,b^ | | | |
| diff | N | Subset for alpha = 0.05 | |
|  |  | 1 | 2 |
| Ac-SKIP -12 | 30 | .1121 |  |
| SKIP -12 | 20 | .1202 |  |
| Contrl | 47 | .1221 |  |
| Ac-SKIP -9 | 64 |  | .1447 |
| SKIP -9 | 43 |  | .1463 |
| Sig. |  | .394 | .998 |
| Means for groups in homogeneous subsets are displayed. | | | |
| a. Uses Harmonic Mean Sample Size = 34.845. | | | |
| b. The group sizes are unequal. The harmonic mean of the group sizes is used. Type I error levels are not guaranteed. | | | |

**Statistical details of result displayed in the figure 3:** Statistical analysis was performed by One-way anova (SPSS 23) with Tukey HSD.

| **Descriptives** | | | | | | | | |
| --- | --- | --- | --- | --- | --- | --- | --- | --- |
| Plateau | | | | | | | | |
|  | N | Mean | Std. Deviation | Std. Error | 95% Confidence Interval for Mean | | Minimum | Maximum |
|  |  |  |  |  | Lower Bound | Upper Bound |  |  |
| Control | 82 | .6907 | .14665 | .01619 | .6585 | .7229 | .38 | .97 |
| Zn | 43 | .8261 | .13217 | .02016 | .7854 | .8668 | .59 | 1.00 |
| Zn+Ac-SKIP 10^(-12)M | 44 | .7150 | .12902 | .01945 | .6757 | .7542 | .43 | 1.00 |
| Zn+SKIP 10^(-12)M | 79 | .6126 | .18931 | .02130 | .5702 | .6550 | .27 | .99 |
| Zn+Ac-SKIP 10^(-9)M | 76 | .6451 | .19602 | .02249 | .6004 | .6899 | .18 | .96 |
| Zn+SKIP 10^(-9)M | 81 | .7272 | .18160 | .02018 | .6870 | .7673 | .29 | .99 |
| Total | 405 | .6912 | .17985 | .00894 | .6737 | .7088 | .18 | 1.00 |

| **ANOVA** | | | | | |
| --- | --- | --- | --- | --- | --- |
| Plateau | | | | | |
|  | Sum of Squares | df | Mean Square | F | Sig. |
| Between Groups | 1.561 | 5 | .312 | 10.829 | .000 |
| Within Groups | 11.507 | 399 | .029 |  |  |
| Total | 13.068 | 404 |  |  |  |

| **Multiple Comparisons** | | | | | | | | | | | |  |
| --- | --- | --- | --- | --- | --- | --- | --- | --- | --- | --- | --- | --- |
| Dependent Variable: | | | | | | | | | | | |  |
| Tukey HSD | | | | | | | | | | | |  |
| (I) diffpl | | | Mean Difference (I-J) | | | Std. Error | | Sig. | | 95% Confidence Interval | |  |
|  |  |  |  |  |  |  |  |  |  | Lower Bound | Upper Bound |  |
| Control | Zn | | -.13541^*^ | | | .03197 | | .000 | | -.2270 | -.0439 |  |
|  | Zn+Ac-SKIP 10^(-12)M | | -.02429 | | | .03174 | | .973 | | -.1152 | .0666 |  |
|  | Zn+SKIP 10^(-12)M | | .07808^*^ | | | .02677 | | .043 | | .0014 | .1547 |  |
|  | Zn+Ac-SKIP 10^(-9)M | | .04554 | | | .02704 | | .543 | | -.0319 | .1230 |  |
|  | Zn+SKIP 10^(-9)M | | -.03649 | | | .02660 | | .744 | | -.1127 | .0397 |  |
| Zn | Control | | .13541^*^ | | | .03197 | | .000 | | .0439 | .2270 |  |
|  | Zn+Ac-SKIP 10^(-12)M | | .11113^*^ | | | .03642 | | .029 | | .0068 | .2154 |  |
|  | Zn+SKIP 10^(-12)M | | .21350^*^ | | | .03218 | | .000 | | .1213 | .3057 |  |
|  | Zn+Ac-SKIP 10^(-9)M | | .18095^*^ | | | .03241 | | .000 | | .0882 | .2738 |  |
|  | Zn+SKIP 10^(-9)M | | .09893^*^ | | | .03204 | | .026 | | .0072 | .1907 |  |
| Zn+Ac-SKIP 10^(-12)M | Control | | .02429 | | | .03174 | | .973 | | -.0666 | .1152 |  |
|  | Zn | | -.11113^*^ | | | .03642 | | .029 | | -.2154 | -.0068 |  |
|  | Zn+SKIP 10^(-12)M | | .10237^*^ | | | .03194 | | .018 | | .0109 | .1938 |  |
|  | Zn+Ac-SKIP 10^(-9)M | | .06983 | | | .03217 | | .254 | | -.0223 | .1619 |  |
|  | Zn+SKIP 10^(-9)M | | -.01220 | | | .03180 | | .999 | | -.1033 | .0789 |  |
| Zn+SKIP 10^(-12)M | Control | | -.07808^*^ | | | .02677 | | .043 | | -.1547 | -.0014 |  |
|  | Zn | | -.21350^*^ | | | .03218 | | .000 | | -.3057 | -.1213 |  |
|  | Zn+Ac-SKIP 10^(-12)M | | -.10237^*^ | | | .03194 | | .018 | | -.1938 | -.0109 |  |
|  | Zn+Ac-SKIP 10^(-9)M | | -.03254 | | | .02729 | | .840 | | -.1107 | .0456 |  |
|  | Zn+SKIP 10^(-9)M | | -.11457^*^ | | | .02685 | | .000 | | -.1915 | -.0377 |  |
| Zn+Ac-SKIP 10^(-9)M | Control | | -.04554 | | | .02704 | | .543 | | -.1230 | .0319 |  |
|  | Zn | | -.18095^*^ | | | .03241 | | .000 | | -.2738 | -.0882 |  |
|  | Zn+Ac-SKIP 10^(-12)M | | -.06983 | | | .03217 | | .254 | | -.1619 | .0223 |  |
|  | Zn+SKIP 10^(-12)M | | .03254 | | | .02729 | | .840 | | -.0456 | .1107 |  |
|  | Zn+SKIP 10^(-9)M | | -.08203^*^ | | | .02712 | | .032 | | -.1597 | -.0044 |  |
| Zn+SKIP 10^(-9)M | Control | | .03649 | | | .02660 | | .744 | | -.0397 | .1127 |  |
|  | Zn | | -.09893^*^ | | | .03204 | | .026 | | -.1907 | -.0072 |  |
|  | Zn+Ac-SKIP 10^(-12)M | | .01220 | | | .03180 | | .999 | | -.0789 | .1033 |  |
|  | Zn+SKIP 10^(-12)M | | .11457^*^ | | | .02685 | | .000 | | .0377 | .1915 |  |
|  | Zn+Ac-SKIP 10^(-9)M | | .08203^*^ | | | .02712 | | .032 | | .0044 | .1597 |  |
| *. The mean difference is significant at the 0.05 level. | | | | | | | | | | | |  |
| **Plateau** | | | | | | | | |  |  |  |  |
| Tukey HSD^a,b^ | | | | | | | | |  |  |  |  |
| diffpl | | N | | Subset for alpha = 0.05 | | | | |  |  |  |  |
|  |  |  |  | 1 | 2 | | 3 | |  |  |  |  |
| Zn+SKIP 10^(-12)M | | 79 | | .6126 |  | |  | |  |  |  |  |
| Zn+Ac-SKIP 10^(-9)M | | 76 | | .6451 | .6451 | |  | |  |  |  |  |
| Control | | 82 | | .6907 | .6907 | |  | |  |  |  |  |
| Zn+Ac-SKIP 10^(-12)M | | 44 | |  | .7150 | |  | |  |  |  |  |
| Zn+SKIP 10^(-9)M | | 81 | |  | .7272 | |  | |  |  |  |  |
| Zn | | 43 | |  |  | | .8261 | |  |  |  |  |
| Sig. | |  | | .108 | .078 | | 1.000 | |  |  |  |  |
| Means for groups in homogeneous subsets are displayed. | | | | | | | | |  |  |  |  |
| a. Uses Harmonic Mean Sample Size = 62.279. | | | | | | | | |  |  |  |  |
| b. The group sizes are unequal. The harmonic mean of the group sizes is used. Type I error levels are not guaranteed. | | | | | | | | |  |  |  |  |

**Statistical details of result displayed in the figure 4B and supplemental figure S4:** Statistical analysis was performed by One-way anova (SPSS 23) with Tukey HSD.

| **Descriptives** | | | | | | | | | |
| --- | --- | --- | --- | --- | --- | --- | --- | --- | --- |
|  | | N | Mean | Std. Deviation | Std. Error | 95% Confidence Interval for Mean | | Minimum | Maximum |
|  |  |  |  |  |  | Lower Bound | Upper Bound |  |  |
| Tubulin | Cont | 15 | .4906 | .23770 | .06137 | .3590 | .6222 | .19 | .97 |
|  | Zn | 18 | .6861 | .11954 | .02818 | .6266 | .7455 | .33 | .80 |
|  | Zn+Ac-SKIP-12M | 15 | .5097 | .25845 | .06673 | .3665 | .6528 | .07 | .91 |
|  | Zn+SKIP-12M | 9 | .3580 | .07539 | .02513 | .3000 | .4159 | .25 | .49 |
|  | Zn+Ac-SKIP-9M | 18 | .4332 | .18067 | .04258 | .3434 | .5231 | .19 | .76 |
|  | Zn+SKIP-9M | 11 | .3372 | .11415 | .03442 | .2605 | .4139 | .11 | .43 |
|  | Total | 86 | .4893 | .21336 | .02301 | .4436 | .5351 | .07 | .97 |
| Actin | Cont | 3 | .4805 | .09614 | .05551 | .2417 | .7193 | .37 | .55 |
|  | Zn | 6 | .4824 | .03554 | .01451 | .4451 | .5197 | .43 | .53 |
|  | Zn+Ac-SKIP-12M | 3 | .4907 | .06048 | .03492 | .3405 | .6410 | .42 | .54 |
|  | Zn+SKIP-12M | 6 | .4879 | .04371 | .01784 | .4421 | .5338 | .42 | .54 |
|  | Zn+Ac-SKIP-9M | 3 | .4493 | .02055 | .01187 | .3982 | .5003 | .43 | .47 |
|  | Zn+SKIP-9M | 3 | .4417 | .07094 | .04095 | .2655 | .6179 | .38 | .52 |
|  | Total | 24 | .4754 | .05110 | .01043 | .4538 | .4969 | .37 | .55 |

| **ANOVA** | | | | | | |
| --- | --- | --- | --- | --- | --- | --- |
|  | | Sum of Squares | df | Mean Square | F | Sig. |
| Tubulin | Between Groups | 1.170 | 5 | .234 | 6.932 | .000 |
|  | Within Groups | 2.700 | 80 | .034 |  |  |
|  | Total | 3.869 | 85 |  |  |  |
| Actin | Between Groups | .007 | 5 | .001 | .512 | .763 |
|  | Within Groups | .053 | 18 | .003 |  |  |
|  | Total | .060 | 23 |  |  |  |

| **Multiple Comparisons** | | | | | | | |
| --- | --- | --- | --- | --- | --- | --- | --- |
| Tukey HSD | | | | | | | |
| Dependent Variable | | | Mean Difference (I-J) | Std. Error | Sig. | 95% Confidence Interval | |
|  |  |  |  |  |  | Lower Bound | Upper Bound |
| Tubulin | Cont | Zn | -.19548^*^ | .06422 | .036 | -.3830 | -.0080 |
|  |  | Zn+Ac-SKIP-12M | -.01905 | .06708 | 1.000 | -.2149 | .1768 |
|  |  | Zn+SKIP-12M | .13263 | .07746 | .528 | -.0935 | .3588 |
|  |  | Zn+Ac-SKIP-9M | .05738 | .06422 | .947 | -.1301 | .2449 |
|  |  | Zn+SKIP-9M | .15341 | .07292 | .296 | -.0595 | .3663 |
|  | Zn | Cont | .19548^*^ | .06422 | .036 | .0080 | .3830 |
|  |  | Zn+Ac-SKIP-12M | .17643 | .06422 | .077 | -.0111 | .3640 |
|  |  | Zn+SKIP-12M | .32811^*^ | .07500 | .001 | .1091 | .5471 |
|  |  | Zn+Ac-SKIP-9M | .25286^*^ | .06123 | .001 | .0741 | .4317 |
|  |  | Zn+SKIP-9M | .34889^*^ | .07030 | .000 | .1436 | .5542 |
|  | Zn+Ac-SKIP-12M | Cont | .01905 | .06708 | 1.000 | -.1768 | .2149 |
|  |  | Zn | -.17643 | .06422 | .077 | -.3640 | .0111 |
|  |  | Zn+SKIP-12M | .15168 | .07746 | .375 | -.0745 | .3778 |
|  |  | Zn+Ac-SKIP-9M | .07643 | .06422 | .840 | -.1111 | .2640 |
|  |  | Zn+SKIP-9M | .17246 | .07292 | .181 | -.0405 | .3854 |
|  | Zn+SKIP-12M | Cont | -.13263 | .07746 | .528 | -.3588 | .0935 |
|  |  | Zn | -.32811^*^ | .07500 | .001 | -.5471 | -.1091 |
|  |  | Zn+Ac-SKIP-12M | -.15168 | .07746 | .375 | -.3778 | .0745 |
|  |  | Zn+Ac-SKIP-9M | -.07525 | .07500 | .916 | -.2942 | .1437 |
|  |  | Zn+SKIP-9M | .02078 | .08257 | 1.000 | -.2203 | .2619 |
|  | Zn+Ac-SKIP-9M | Cont | -.05738 | .06422 | .947 | -.2449 | .1301 |
|  |  | Zn | -.25286^*^ | .06123 | .001 | -.4317 | -.0741 |
|  |  | Zn+Ac-SKIP-12M | -.07643 | .06422 | .840 | -.2640 | .1111 |
|  |  | Zn+SKIP-12M | .07525 | .07500 | .916 | -.1437 | .2942 |
|  |  | Zn+SKIP-9M | .09603 | .07030 | .747 | -.1093 | .3013 |
|  | Zn+SKIP-9M | Cont | -.15341 | .07292 | .296 | -.3663 | .0595 |
|  |  | Zn | -.34889^*^ | .07030 | .000 | -.5542 | -.1436 |
|  |  | Zn+Ac-SKIP-12M | -.17246 | .07292 | .181 | -.3854 | .0405 |
|  |  | Zn+SKIP-12M | -.02078 | .08257 | 1.000 | -.2619 | .2203 |
|  |  | Zn+Ac-SKIP-9M | -.09603 | .07030 | .747 | -.3013 | .1093 |
| Actin | Cont | Zn | -.00189 | .03822 | 1.000 | -.1233 | .1196 |
|  |  | Zn+Ac-SKIP-12M | -.01022 | .04413 | 1.000 | -.1505 | .1300 |
|  |  | Zn+SKIP-12M | -.00741 | .03822 | 1.000 | -.1289 | .1140 |
|  |  | Zn+Ac-SKIP-9M | .03127 | .04413 | .978 | -.1090 | .1715 |
|  |  | Zn+SKIP-9M | .03883 | .04413 | .947 | -.1014 | .1791 |
|  | Zn | Cont | .00189 | .03822 | 1.000 | -.1196 | .1233 |
|  |  | Zn+Ac-SKIP-12M | -.00833 | .03822 | 1.000 | -.1298 | .1131 |
|  |  | Zn+SKIP-12M | -.00552 | .03120 | 1.000 | -.1047 | .0936 |
|  |  | Zn+Ac-SKIP-9M | .03316 | .03822 | .949 | -.0883 | .1546 |
|  |  | Zn+SKIP-9M | .04072 | .03822 | .889 | -.0807 | .1622 |
|  | Zn+Ac-SKIP-12M | Cont | .01022 | .04413 | 1.000 | -.1300 | .1505 |
|  |  | Zn | .00833 | .03822 | 1.000 | -.1131 | .1298 |
|  |  | Zn+SKIP-12M | .00281 | .03822 | 1.000 | -.1186 | .1243 |
|  |  | Zn+Ac-SKIP-9M | .04149 | .04413 | .930 | -.0987 | .1817 |
|  |  | Zn+SKIP-9M | .04905 | .04413 | .870 | -.0912 | .1893 |
|  | Zn+SKIP-12M | Cont | .00741 | .03822 | 1.000 | -.1140 | .1289 |
|  |  | Zn | .00552 | .03120 | 1.000 | -.0936 | .1047 |
|  |  | Zn+Ac-SKIP-12M | -.00281 | .03822 | 1.000 | -.1243 | .1186 |
|  |  | Zn+Ac-SKIP-9M | .03868 | .03822 | .908 | -.0828 | .1601 |
|  |  | Zn+SKIP-9M | .04624 | .03822 | .826 | -.0752 | .1677 |
|  | Zn+Ac-SKIP-9M | Cont | -.03127 | .04413 | .978 | -.1715 | .1090 |
|  |  | Zn | -.03316 | .03822 | .949 | -.1546 | .0883 |
|  |  | Zn+Ac-SKIP-12M | -.04149 | .04413 | .930 | -.1817 | .0987 |
|  |  | Zn+SKIP-12M | -.03868 | .03822 | .908 | -.1601 | .0828 |
|  |  | Zn+SKIP-9M | .00756 | .04413 | 1.000 | -.1327 | .1478 |
|  | Zn+SKIP-9M | Cont | -.03883 | .04413 | .947 | -.1791 | .1014 |
|  |  | Zn | -.04072 | .03822 | .889 | -.1622 | .0807 |
|  |  | Zn+Ac-SKIP-12M | -.04905 | .04413 | .870 | -.1893 | .0912 |
|  |  | Zn+SKIP-12M | -.04624 | .03822 | .826 | -.1677 | .0752 |
|  |  | Zn+Ac-SKIP-9M | -.00756 | .04413 | 1.000 | -.1478 | .1327 |
| *. The mean difference is significant at the 0.05 level. | | | | | | | |

| **Tubulin** | | | |
| --- | --- | --- | --- |
| Tukey HSD^a,b^ | | | |
| difff | N | Subset for alpha = 0.05 | |
|  |  | 1 | 2 |
| Zn+SKIP-9M | 11 | .3372 |  |
| Zn+SKIP-12M | 9 | .3580 |  |
| Zn+Ac-SKIP-9M | 18 | .4332 |  |
| Cont | 15 | .4906 | .4906 |
| Zn+Ac-SKIP-12M | 15 | .5097 | .5097 |
| Zn | 18 |  | .6861 |
| Sig. |  | .157 | .075 |
| Means for groups in homogeneous subsets are displayed. | | | |
| a. Uses Harmonic Mean Sample Size = 13.439. | | | |
| b. The group sizes are unequal. The harmonic mean of the group sizes is used. Type I error levels are not guaranteed. | | | |

| **Actin** | | |
| --- | --- | --- |
| Tukey HSD^a,b^ | | |
| difff | N | Subset for alpha = 0.05 |
|  |  | 1 |
| Zn+SKIP-9M | 3 | .4417 |
| Zn+Ac-SKIP-9M | 3 | .4493 |
| Cont | 3 | .4805 |
| Zn | 6 | .4824 |
| Zn+SKIP-12M | 6 | .4879 |
| Zn+Ac-SKIP-12M | 3 | .4907 |
| Sig. |  | .823 |
| Means for groups in homogeneous subsets are displayed. | | |
| a. Uses Harmonic Mean Sample Size = 3.600. | | |
| b. The group sizes are unequal. The harmonic mean of the group sizes is used. Type I error levels are not guaranteed. | | |

**Statistical details of result displayed in the figure 4C (IP: EB1) and 4D (IP: Tau):** Statistical analysis was performed by One-way anova (SPSS 23) with LSD HSD.

| Descriptives | |  |  |  |  |  |  |  |  |
| --- | --- | --- | --- | --- | --- | --- | --- | --- | --- |
|  |  | N | Mean | Std. Deviation | Std. Error | 95% Confidence Interval for Mean | | Minimum | Maximum |
|  |  |  |  |  |  | Lower Bound | Upper Bound | |  |
| IPeb1IBeb1 | Cont | 3 | 39.36 | 7.74111 | 4.46933 | 20.13 | 58.59 | 31 | 46.28 |
|  | SKIP | 3 | 408.6333 | 231.6369 | 133.7356 | -166.785 | 984.0512 | 210.93 | 663.5 |
|  | Ac-SKIP | 3 | 141.4533 | 124.3681 | 71.80397 | -167.494 | 450.4009 | 58.23 | 284.42 |
|  | Total | 9 | 196.4822 | 211.1093 | 70.36976 | 34.2093 | 358.7552 | 31 | 663.5 |
| IPeb1IBtau | Cont | 3 | 8.6133 | 1.16363 | 0.67182 | 5.7227 | 11.5039 | 7.54 | 9.85 |
|  | SKIP | 3 | 49.7167 | 6.57888 | 3.79832 | 33.3738 | 66.0595 | 44.68 | 57.16 |
|  | Ac-SKIP | 3 | 47.5 | 32.19855 | 18.58984 | -32.4856 | 127.4856 | 12.67 | 76.18 |
|  | Total | 9 | 35.2767 | 25.90689 | 8.63563 | 15.3629 | 55.1905 | 7.54 | 76.18 |
| IPeb1IBtubulin | Cont | 3 | 14.95 | 1.7476 | 1.00898 | 10.6087 | 19.2913 | 13.15 | 16.64 |
|  | SKIP | 3 | 18.4667 | 9.58682 | 5.53495 | -5.3483 | 42.2816 | 7.97 | 26.76 |
|  | Ac-SKIP | 2 | 9.54 | 4.72347 | 3.34 | -32.8987 | 51.9787 | 6.2 | 12.88 |
|  | Total | 8 | 14.9163 | 6.63176 | 2.34468 | 9.372 | 20.4605 | 6.2 | 26.76 |
| IPtauIBeb1 | Cont | 3 | 29.26 | 1.58606 | 0.91571 | 25.32 | 33.2 | 27.56 | 30.7 |
|  | SKIP | 3 | 247.0933 | 116.0011 | 66.97325 | -41.0693 | 535.256 | 160.41 | 378.87 |
|  | Ac-SKIP | 3 | 206.65 | 97.04258 | 56.02756 | -34.4171 | 447.7171 | 104.06 | 296.98 |
|  | Total | 9 | 161.0011 | 125.6515 | 41.88382 | 64.4169 | 257.5854 | 27.56 | 378.87 |
| IPtauIBtau | Cont | 3 | 238.4 | 22.54768 | 13.01791 | 182.3885 | 294.4115 | 219.07 | 263.17 |
|  | SKIP | 3 | 347.5833 | 100.2202 | 57.86215 | 98.6226 | 596.5441 | 240 | 438.3 |
|  | Ac-SKIP | 3 | 234.3233 | 16.13987 | 9.31836 | 194.2297 | 274.417 | 220.68 | 252.14 |
|  | Total | 9 | 273.4356 | 76.15068 | 25.38356 | 214.901 | 331.9701 | 219.07 | 438.3 |
| IPtauIBtubulin | Cont | 3 | 12.0467 | 5.72715 | 3.30657 | -2.1804 | 26.2737 | 5.5 | 16.13 |
|  | SKIP | 3 | 47.9933 | 17.03768 | 9.83671 | 5.6694 | 90.3173 | 32.5 | 66.24 |
|  | Ac-SKIP | 3 | 51.1733 | 19.70593 | 11.37722 | 2.2211 | 100.1256 | 34.32 | 72.84 |
|  | Total | 9 | 37.0711 | 23.06508 | 7.68836 | 19.3417 | 54.8005 | 5.5 | 72.84 |

| ANOVA |  |  |  |  |  |  |
| --- | --- | --- | --- | --- | --- | --- |
|  |  | Sum of Squares | df | Mean Square | F | Sig. |
| IPeb1IBeb1 | Between Groups | 218171 | 2 | 109085.5 | 4.73 | 0.058 |
|  | Within Groups | 138366 | 6 | 23061 |  |  |
|  | Total | 356537 | 8 |  |  |  |
| IPeb1IBtau | Between Groups | 3206.57 | 2 | 1603.285 | 4.448 | 0.065 |
|  | Within Groups | 2162.765 | 6 | 360.461 |  |  |
|  | Total | 5369.336 | 8 |  |  |  |
| IPeb1IBtubulin | Between Groups | 95.628 | 2 | 47.814 | 1.126 | 0.395 |
|  | Within Groups | 212.233 | 5 | 42.447 |  |  |
|  | Total | 307.861 | 7 |  |  |  |
| IPtauIBtau | Between Groups | 80554.24 | 2 | 40277.12 | 5.282 | 0.048 |
|  | Within Groups | 45752.06 | 6 | 7625.342 |  |  |
|  | Total | 126306.3 | 8 |  |  |  |
| IPtauIBeb1 | Between Groups | 24765.45 | 2 | 12382.72 | 3.436 | 0.101 |
|  | Within Groups | 21625.96 | 6 | 3604.326 |  |  |
|  | Total | 46391.41 | 8 |  |  |  |
| IPtauIBtubulin | Between Groups | 2833.171 | 2 | 1416.586 | 5.974 | 0.037 |
|  | Within Groups | 1422.813 | 6 | 237.135 |  |  |
|  | Total | 4255.984 | 8 |  |  |  |

| Multiple Comparisons | | |  |  |  |  |  |
| --- | --- | --- | --- | --- | --- | --- | --- |
| LSD |  |  |  |  |  |  |  |
| Dependent Variable | (I) diff | (J) diff | Mean Difference (I-J) | Std. Error | Sig. | 95% Confidence Interval | |
|  |  |  |  |  |  | Lower Bound | Upper Bound |
| IPeb1IBeb1 | Cont | SKIP | -369.27333* | 123.9919 | 0.025 | -672.671 | -65.876 |
|  |  | Ac-SKIP | -102.093 | 123.9919 | 0.442 | -405.491 | 201.304 |
|  | SKIP | Cont | 369.27333* | 123.9919 | 0.025 | 65.876 | 672.6706 |
|  |  | Ac-SKIP | 267.18 | 123.9919 | 0.075 | -36.2173 | 570.5773 |
|  | Ac-SKIP | Cont | 102.0933 | 123.9919 | 0.442 | -201.304 | 405.4906 |
|  |  | SKIP | -267.18 | 123.9919 | 0.075 | -570.577 | 36.2173 |
| IPeb1IBtau | Cont | SKIP | -41.10333* | 15.50185 | 0.038 | -79.035 | -3.1717 |
|  |  | Ac-SKIP | -38.88667* | 15.50185 | 0.046 | -76.8183 | -0.955 |
|  | SKIP | Cont | 41.10333* | 15.50185 | 0.038 | 3.1717 | 79.035 |
|  |  | Ac-SKIP | 2.21667 | 15.50185 | 0.891 | -35.715 | 40.1483 |
|  | Ac-SKIP | Cont | 38.88667* | 15.50185 | 0.046 | 0.955 | 76.8183 |
|  |  | SKIP | -2.21667 | 15.50185 | 0.891 | -40.1483 | 35.715 |
| IPeb1IBtubulin | Cont | SKIP | -3.51667 | 5.31957 | 0.538 | -17.191 | 10.1577 |
|  |  | Ac-SKIP | 5.41 | 5.94746 | 0.405 | -9.8784 | 20.6984 |
|  | SKIP | Cont | 3.51667 | 5.31957 | 0.538 | -10.1577 | 17.191 |
|  |  | Ac-SKIP | 8.92667 | 5.94746 | 0.194 | -6.3618 | 24.2151 |
|  | Ac-SKIP | Cont | -5.41 | 5.94746 | 0.405 | -20.6984 | 9.8784 |
|  |  | SKIP | -8.92667 | 5.94746 | 0.194 | -24.2151 | 6.3618 |
| IPtauIBteb1 | Cont | SKIP | -217.83333* | 71.2991 | 0.022 | -392.296 | -43.3707 |
|  |  | Ac-SKIP | -177.39000* | 71.2991 | 0.047 | -351.853 | -2.9274 |
|  | SKIP | Cont | 217.83333* | 71.2991 | 0.022 | 43.3707 | 392.2959 |
|  |  | Ac-SKIP | 40.44333 | 71.2991 | 0.591 | -134.019 | 214.9059 |
|  | Ac-SKIP | Cont | 177.39000* | 71.2991 | 0.047 | 2.9274 | 351.8526 |
|  |  | SKIP | -40.4433 | 71.2991 | 0.591 | -214.906 | 134.0193 |
| IPtauIBtau | Cont | SKIP | -109.183 | 49.01922 | 0.068 | -229.129 | 10.7624 |
|  |  | Ac-SKIP | 4.07667 | 49.01922 | 0.936 | -115.869 | 124.0224 |
|  | SKIP | Cont | 109.1833 | 49.01922 | 0.068 | -10.7624 | 229.1291 |
|  |  | Ac-SKIP | 113.26 | 49.01922 | 0.06 | -6.6857 | 233.2057 |
|  | Ac-SKIP | Cont | -4.07667 | 49.01922 | 0.936 | -124.022 | 115.8691 |
|  |  | SKIP | -113.26 | 49.01922 | 0.06 | -233.206 | 6.6857 |
| IPtauIBtubulin | Cont | SKIP | -35.94667* | 12.5734 | 0.029 | -66.7127 | -5.1807 |
|  |  | Ac-SKIP | -39.12667* | 12.5734 | 0.021 | -69.8927 | -8.3607 |
|  | SKIP | Cont | 35.94667* | 12.5734 | 0.029 | 5.1807 | 66.7127 |
|  |  | Ac-SKIP | -3.18 | 12.5734 | 0.809 | -33.946 | 27.586 |
|  | Ac-SKIP | Cont | 39.12667* | 12.5734 | 0.021 | 8.3607 | 69.8927 |
|  |  | SKIP | 3.18 | 12.5734 | 0.809 | -27.586 | 33.946 |
| * The mean difference is significant at the 0.05 level. | | | | | |  |  |
